# Bias-Driven Hot-Correlated Electrons in the Two-Dimensional Ferromagnet Fe_4_GeTe_2_

**DOI:** 10.1021/acs.nanolett.6c02700

**Published:** 2026-09-09

**Authors:** Declan Nell, Stefano Sanvito, Andrea Droghetti

**Affiliations:** † School of Physics and CRANN Institute, 8809 Trinity College Dublin, The University of Dublin, Dublin D02 PN40, Ireland; ‡ Department of Molecular Sciences and Nanosystems, 19047 Ca’ Foscari University of Venice, via Torino 155, Mestre, 30170 Venice, Italy

**Keywords:** Quantum transport, nonequilibrium transport, dynamical mean-field theory, nonequilibrium Green’s functions, electronic correlations, spin transport, van der Waals ferromagnets

## Abstract

Electronic correlations can strongly influence the properties of magnetic materials, yet their role in charge transport in devices under operating conditions remains largely unexplored. Here, we show that an applied bias voltage can drive a breakdown of quasiparticle coherence in the prototypical two-dimensional ferromagnet Fe_4_GeTe_2_. Using a first-principles framework combining density functional theory, dynamical mean-field theory, and nonequilibrium Green’s functions, we predict a voltage-induced transition from coherent, nearly half-metallic transport to a nonequilibrium regime dominated by strong inelastic scattering of charge carriers and collective electron–hole excitations. This emergent “hot-correlated electron” regime exhibits distinct spectral and transport signatures accessible in experiments. More generally, our results reveal how an applied bias reshapes electronic correlations in ferromagnets, modifying the current's spin-polarization and potentially impacting spintronic device performance.

## Graphical abstract

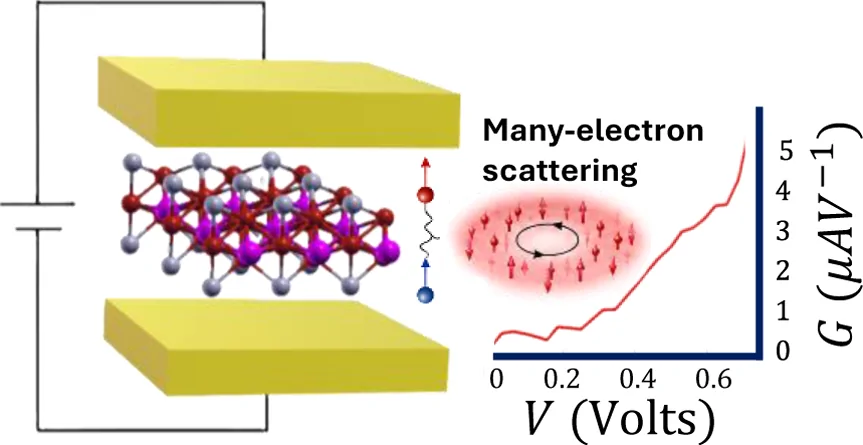

Charge transport in electronic and spintronic devices is commonly treated as coherent, even under applied bias. Yet, a finite voltage drives the system out of equilibrium and can renormalize electron–electron interactions, potentially modifying the electronic structure and device functionality.^1^

van der Waals ferromagnets such as Fe_*n*_Ge­(Ga)­Te_2_ (*n* = 3–5)^2–5^ provide an ideal platform to explore these effects, owing to the coexistence of itinerant and localized electrons and the resulting enhanced electronic correlations.^6–8^ These materials exhibit robust magnetism near or above room temperature^4,5,9–12^ down to the monolayer limit^10,13,14^ and have demonstrated strong spin filtering and large tunneling magnetoresistance in magnetic tunnel junctions,^15–26^ underscoring their promise for spintronic applications. However, the effect of many-body interactions on transport under finite bias remains largely unexplored. From a technological perspective, this raises the open question of whether electronic correlations can fundamentally limit the performance of tunnel junctions, for instance, by degrading spin filtering.

Here, we investigate an Fe_4_GeTe_2_ monolayer contacted by metallic electrodes under finite bias [Figure 1(a)] and uncover a bias-driven crossover from coherent, nearly half-metallic transport to an emergent nonequilibrium regime dominated by strong many-body interactions. Using a recently introduced framework for correlated electron transport,^27^ we predict that, at high bias, inelastic scattering of charge carriers with electron–hole excitations leads to enhanced quasiparticle damping and the emergence of incoherent spectral weights. We term this the “hot-correlated electron” regime. It produces clear signatures in the momentum-resolved spectral function [Figure 1(b),(c)] and nonlinear features in the spin-resolved differential conductance [Figure 1(d)] at experimentally accessible voltages. Beyond their fundamental relevance, our results demonstrate that many-body scattering can also qualitatively reshape spin-dependent transport, modifying the current’s spin-polarization, thereby potentially impacting spintronic device performance.

**Figure 1:**
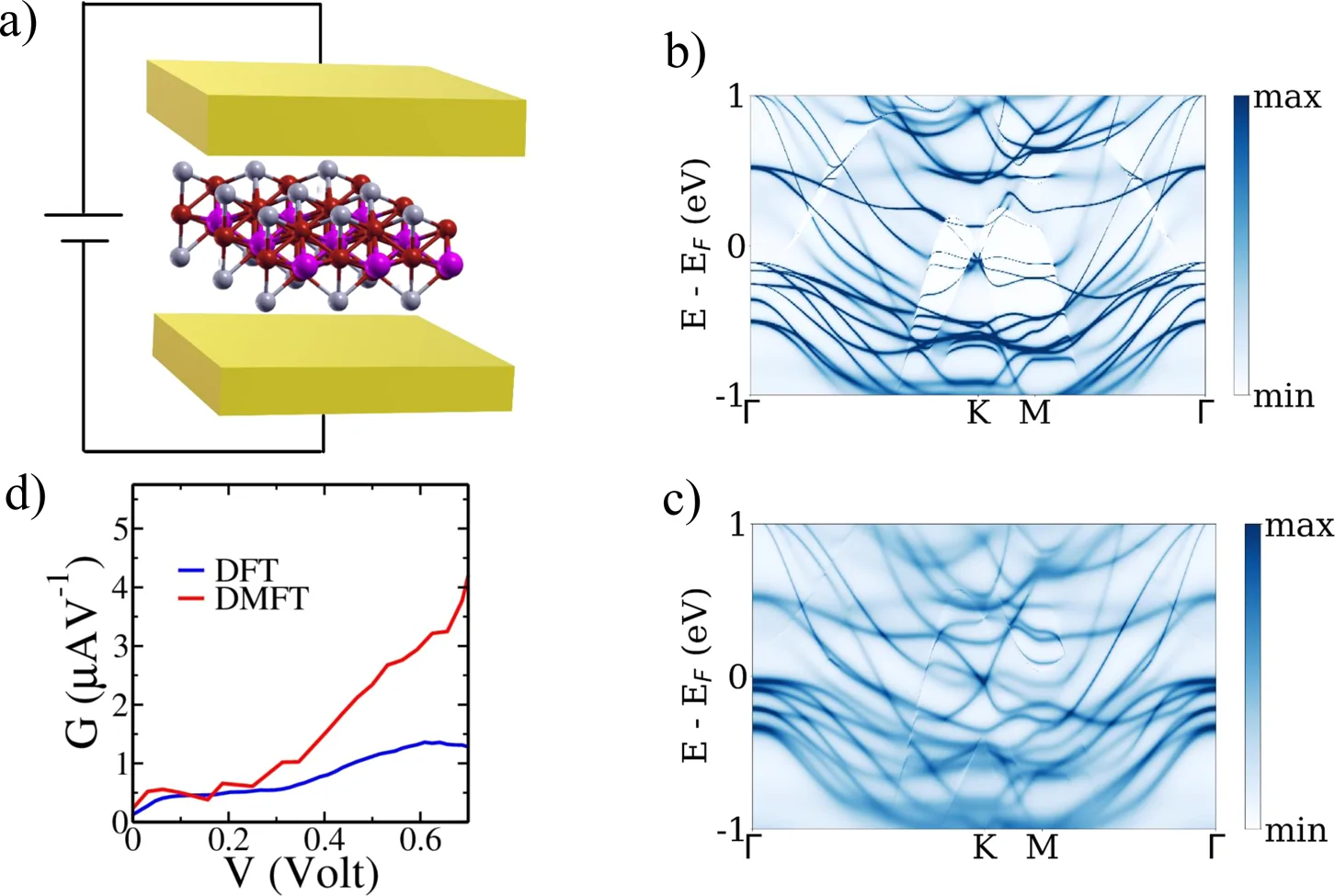
(a) Fe_4_GeTe_2_ monolayer device, where Fe, Te, and Ge are shown as red, gray, and magenta spheres, respectively. (b) and (c) ***k***-resolved Fe 3d spectral functions at 0 and 0.75 V calculated within DMFT. At zero bias, the spectral features near *E*_F_ remain relatively well-defined, retaining a band-like character, while, at finite bias, the bands near Γ flatten and smear into faint traces superimposed on an incoherent background. The apparent discontinuity, visible as a light region in the center of the spectrum, arises from hybridization with the bands of the electrodes, as discussed in Sec. S10 of the SI. (d) Spin-down differential conductance, *G* = d*I* ^*↓*^/d*V*, as a function of the applied bias *V*, calculated with DFT and DMFT, showing that correlation effects (DMFT) lead to a rapid increase of *G* at *V* of ∼0.3 V.

To model realistic devices, we consider a vertical two-probe junction comprising an Fe_4_GeTe_2_ monolayer contacted by metallic electrodes, as shown in Figure 1(a) [see Sec. S2 in the Supporting Information (SI) for further details]. A bias voltage *V* shifts the electrode chemical potentials, μ_L(R)_ = *E*_F_ ± *eV*/2, inducing current perpendicular to the layer. The difference μ_L_ – μ_R_ defines the bias window, i.e., the energy range over which electronic states are driven out of equilibrium and contribute to transport.

Our computational framework combines density functional theory (DFT), dynamical mean-field theory (DMFT),^28,29^ and nonequilibrium Green’s functions (NEGFs)^30–35^ into a self-consistent description of steady-state transport in correlated systems,^27,36,37^ as summarized in Sec. S3 of the SI. DFT provides the material-specific electronic structure; DMFT captures local dynamical correlations in the Fe 3d orbitals, and NEGF enables the treatment of finite bias. This approach goes beyond previous studies of correlated transport, which have been restricted to linear response,^100–42^ while finite-bias effects have been addressed primarily in model systems.^43–46^

Electronic correlations enter through the many-body self-energy Σ^σ^(*E*), which encodes interaction-induced renormalization and scattering processes. Within DMFT, Σ^σ^(*E*) is local and atom-dependent. Out of equilibrium, it acquires distinct retarded and lesser components: the retarded part Σ^σ*r*^(*E*) governs spectral properties, including energy renormalization and quasiparticle lifetimes, while the lesser component Σ^σ<^(*E*) determines the nonequilibrium occupation of electronic states. These components are linked by the fluctuation–dissipation theorem, $\Sigma^{\sigma <}\left(E\right)=-f\left(E\right)\left[\Sigma^{\sigma r}\left(E\right)-\Sigma^{\sigma r}{\left(E\right)}^{†}\right]$ in equilibrium, where *f*(*E*) is the Fermi function.^30^ Deviations from this relation under finite bias provide a direct measure of nonequilibrium correlation effects.

To establish a baseline for correlated transport, we first examine the electronic structure of Fe_4_GeTe_2_ at zero bias for a collinear spin configuration. The Fe-projected density of states (PDOS) is shown in Figure 2(a). Within DFT, which provides an effective single-particle description of the system, the spin-up PDOS exhibits a broad peak crossing *E*_F_, whereas the spin-down channel displays a pseudogap of ∼0.65 eV, bounded by peaks at −0.3 and +0.35 eV. The latter in fact consists of a two-peak structure at ∼0.35 and 0.55 eV associated with inequivalent Fe atoms (see Supporting Information of ref 41). The PDOS is broadened by hybridization with the metallic leads. Overall, DFT predicts highly spin-polarized electronic states at the Fermi level, consistent with the results of previous studies.^41,47^ This picture is indirectly supported by the very large tunneling magnetoresistance ratios reported for magnetic tunnel junctions based on Fe_4_GeTe_2_ and related compounds, using a variety of tunnel barriers.^15,16,19,21,22^

**Figure 2:**
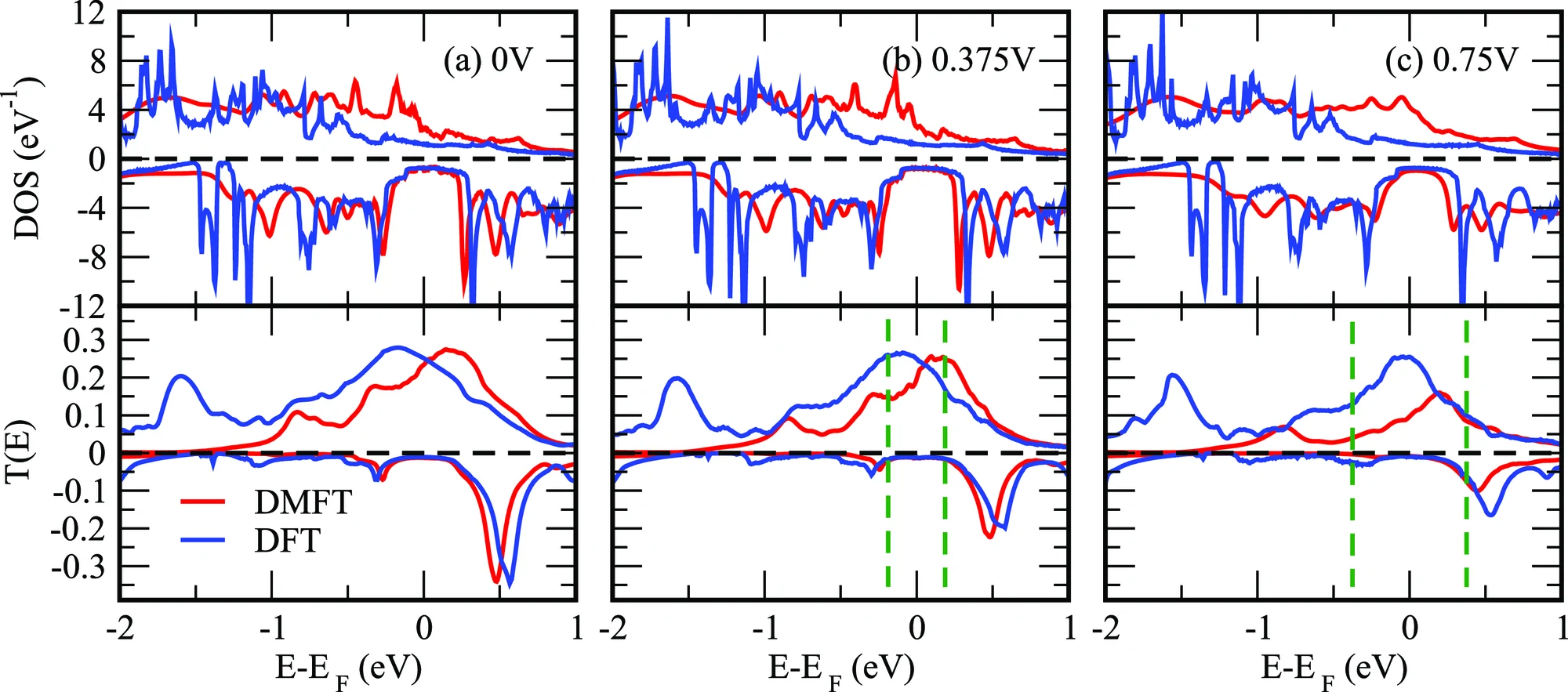
Fe 3d-PDOS and transmission coefficient at (a) 0 V, (b) 0.375 V, and (c) 0.75 V, calculated with DFT (red line) and DMFT (blue line). Spin-up (down) values are shown as positive (negative). The green vertical bars mark the bias window.

Including dynamical correlations via DMFT leads to a redistribution of spectral weight. The real part of the retarded self-energy, Re­[Σ^σ*r*^(*E*)] [Figure 3(a)], shifts spin-up states to higher energies and induces a moderate renormalization, resulting in a sharpening of the PDOS near *E*_F_ and an effective mass enhancement of ∼1.2. In contrast, the spin-down channel is only weakly affected, owing to its lower PDOS near *E*_F_, resulting in a smaller self-energy [Figure 3(b)] and a modest reduction of the pseudogap, accompanied by an effective mass enhancement of ∼1.1. Thus, dynamical correlations do not drastically modify the DFT magnetic state (see Sec. S8 of the SI), and the system retains its high spin polarization.

**Figure 3:**
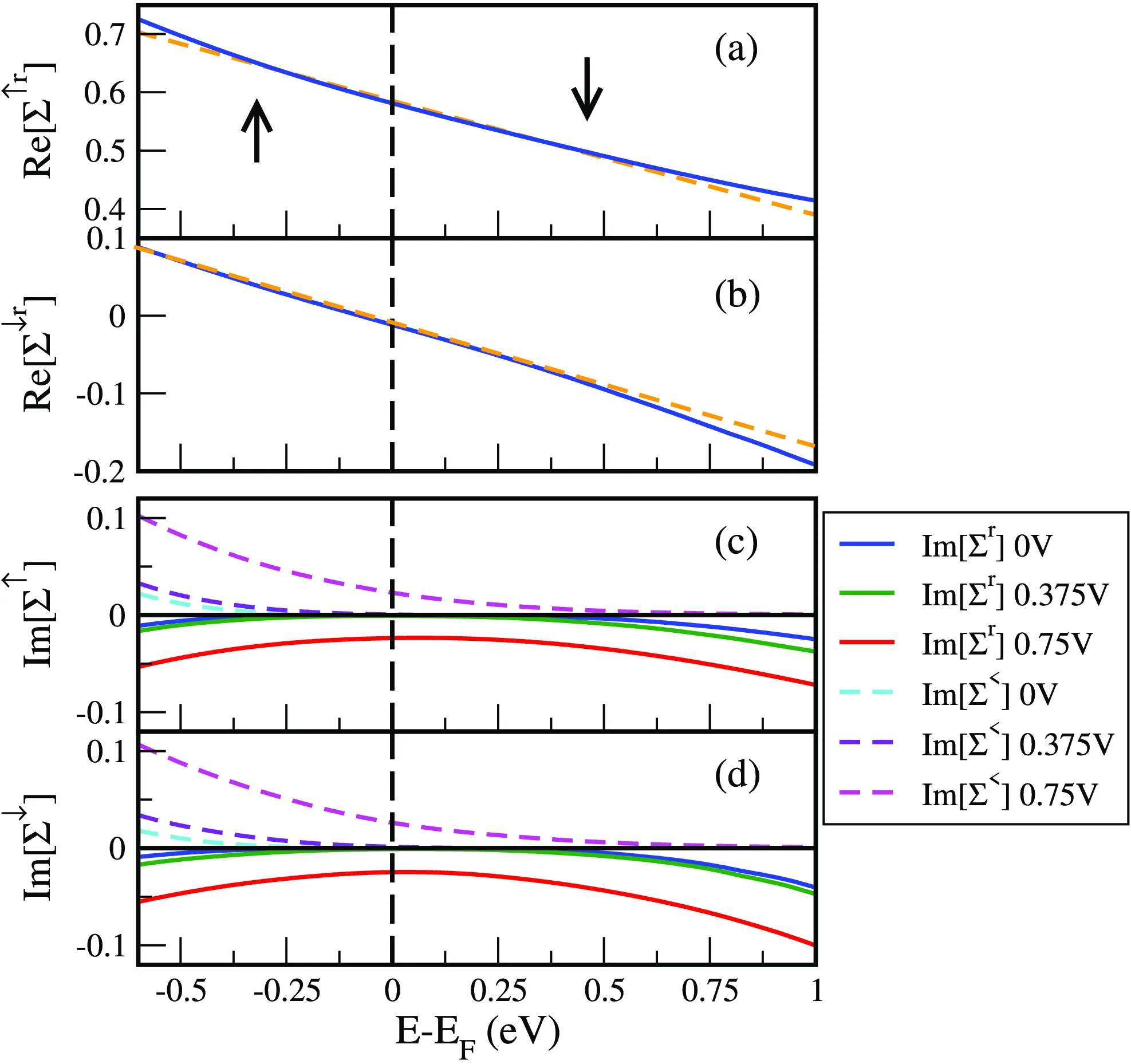
Self-energies as a function of energy and bias. Top panels: real part of the retarded DMFT self-energy at zero bias and averaged over the Fe 3d orbitals for the (a) spin-up and (b) spin-down channels. The orange line shows a linear fit around *E*_F_ with deviations revealing kinks, indicated by the black arrows. Bottom panels: the imaginary part of the retarded and lesser self-energy, averaged over the Fe 3d orbitals, for (c) spin-up and (d) spin-down channels at biases of 0, 0.375, and 0.75 V.

Yet, the many-body self-energy reveals distinct signatures of interaction effects. In both spin channels, Re­[Σ^σ*r*^(*E*)] exhibits kinks, indicated by the arrows in Figure 3(a), at energies corresponding to the spin-down peaks in the PDOS. This reflects enhanced interaction of electrons in one spin channel with electron–hole pairs forming collective excitations in the opposite one. Such features are inherently many-body in nature, and they have previously been reported, for instance, at Fe surfaces.^48^

The imaginary part of the retarded self-energy [Figure 3(c),(d)] follows Fermi-liquid behavior near *E*_F_, $Im\left[\Sigma^{\sigma r}\left(E\right)\right]\propto -{\left(E-E_{F}\right)}^{2}$ , indicating coherent quasiparticles at low energy. At higher energies, deviations from this behavior coincide with the kinks in Re­[Σ^σ*r*^(*E*)], reflecting enhanced scattering due to electron–hole excitations.^49^ The lesser self-energy [cyan dashed line in Figure 3(c),(d)] satisfies the fluctuation–dissipation theorem, confirming that equilibrium occupations are correctly reproduced.

Applying a finite bias drives the system out of equilibrium. Within DFT, the PDOS exhibits only minor bias-induced changes, namely, a slight sharpening of the spin-up states near *E*_F_ and a modest broadening of the spin-down channel. In contrast, DMFT predicts a pronounced, bias-dependent restructuring of the electronic spectrum, as shown in Figure 2(b),(c), and we can identify distinct regimes.

At low bias, *V* ≲ 0.25 V, the PDOS remains largely unchanged. The bias window spans the spin-up peak and the spin-down pseudogap near *E*_F_, while Im­[Σ^σ*r*^(*E*)] retains its Fermi-liquid character, meaning that the excitations near *E*_F_ remain coherent and quasiparticle-like.

For *V* > 0.25 V, the bias window begins to overlap with the tail of the spin-down valence peak [Figure 2(b)]. Correspondingly, Im­[Σ^*σr*^(*E*)] near *E*_F_ increases, signaling enhanced damping of electronic excitations.

At higher bias (*V* ≳ 0.5 V), the bias window extends across the spin-down peak [Figure 2(c)], substantially enhancing coupling to electron–hole excitations and marking the onset of a genuinely nonequilibrium many-body regime. The PDOS is strongly reshaped, particularly in the spin-up channel, where spectral features broaden and smoothen, while the spin-down pseudogap is partially reduced. This behavior reflects the emergence of a sizable Im­[Σ^σ*r*^(*E*)] at *E*_F_ [red line in Figure 3(c),(d)], indicating the breakdown of coherent quasiparticles. Simultaneously, Σ^σ<^(*E*) [magenta dashed line in Figure 3(c),(d)] no longer satisfies the fluctuation–dissipation theorem, demonstrating that the electronic distribution deviates from a Fermi–Dirac form.

The different regimes can be further distinguished by analyzing the spectral function, *A*(*E*, ***k***), which represents the energy- and momentum-resolved electronic spectrum. At zero bias, the DMFT spectral features near *E*_F_ remain relatively well-defined, retaining a band-like character [Figure 1(b)] (see Sec. S3 of the SI for a comparison with DFT). *A*(*E*, ***k***) shows characteristic parabolic bands crossing *E*_F_ near the *K* point of the hexagonal Brillouin zone^41^ [Figure 1(b)]. Nearly flat bands appear around Γ, originating from Fe $d_{z^{2}}$ orbitals that underpin the correlated and magnetic character of the system.^50^ We note that the apparent discontinuity at the center of the spectrum originates from the hybridization of the Fe_4_GeTe_2_ and electrode states (see Sec. S10 of the SI) and is therefore not an intrinsic feature of Fe_4_GeTe_2_.

As the bias increases and the system enters the intermediate nonequilibrium regime, spectral broadening becomes more pronounced across the Brillouin zone, reflecting shorter excitation lifetimes. Finally, in the high-bias, non-Fermi-liquid regime—for example, at *V* = 0.75 V [Figure 1(c)]—the bands near Γ further flatten and smear into faint traces superimposed on an incoherent background. Similar spectral changes have been reported in high-temperature DMFT calculations.^51^ By analogy, we attribute this behavior to energy injection from the applied bias into the Fe_4_GeTe_2_ layer, effectively heating the electronic subsystem and enhancing scattering processes that destroy quasiparticle coherence. Importantly, such nonequilibrium spectral features and, in particular, the bias-induced broadening and loss of the state near Γ provide a direct spectroscopic fingerprint of the hot-correlated regime that can be probed via nano-ARPES.^52–54^

The signatures of the “hot-correlated electron” regime can also be observed in the transport properties, as shown in Figure 4. The charge current is calculated using the Meir–Wingreen formalism.^55^ Within DFT, this reduces to the finite-bias generalization of the Landauer–Büttiker approach:^56^ transport is coherent and spin-conserving, characterized by the spin-resolved transmission coefficient *T* ^σ^(*E*) through the device. The total current can then be decomposed into spin-resolved contributions *I* ^σ^, obtained by integrating *T* ^σ^(*E*) over the bias window (see Sec. S3-D in the SI).

**Figure 4:**
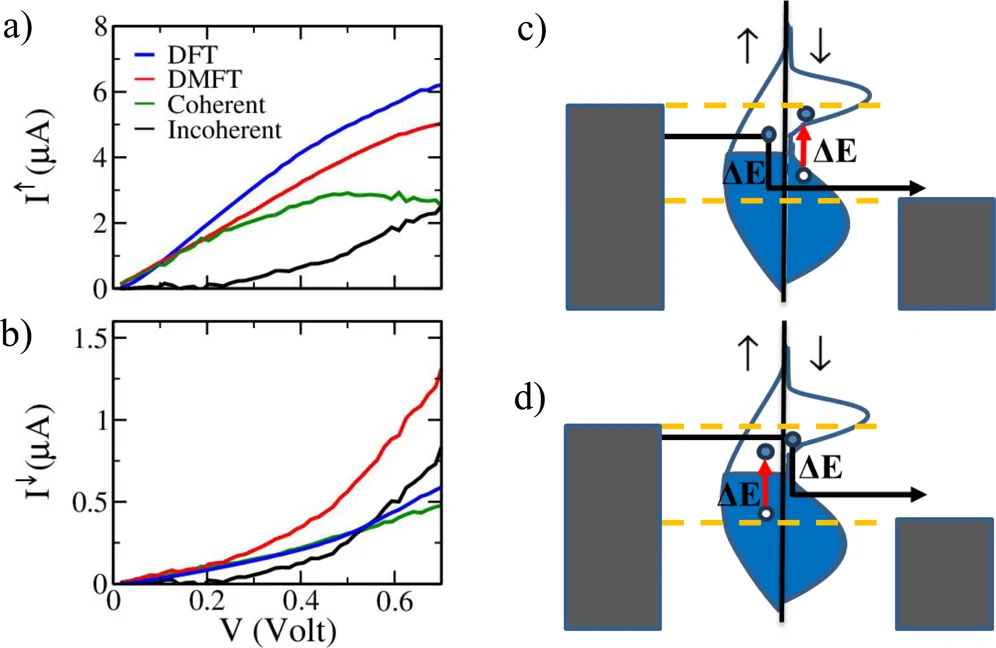
(a, b) Charge currents for the spin-up and spin-down channels as a function of the applied bias *V*. Note the different scale on the vertical axis of the two plots. (c, d) Schematic illustration of dominant inelastic scattering processes for spin-up and spin-down electrons. Dark gray rectangles represent the electrode Fermi boxes with the schematic Fe PDOS shown in between; filled states are shaded in blue. The orange lines mark the bias window. Electrons and holes are represented by filled and empty dots, respectively. In (c), a spin-up electron coming from the left electrode scatters off a spin-down electron in Fe_4_GeTe_2_, generating an electron–hole excitation and transferring part of its energy, Δ*E*, before tunneling into an empty state in the right lead. In (d), a spin-down electron coming from the left lead enters the spin-down conduction band of Fe_4_GeTe_2_, transferring part of its energy, Δ*E*, to an electron–hole excitation in the spin-up channel, before tunneling into an empty state in the right lead.

The transmission coefficient closely follows the Fe PDOS [bottom panels of Figure 2], particularly in the spin-up channel. In contrast, spin-down transmission is dominated by the conduction peak near 0.55 eV, arising from the hybridization between the Fe and the more delocalized Te states that couple to the electrodes, whereas the valence states below −0.25 eV are much more localized and therefore contribute only weakly to the transmission (see Sec. S7 of the SI). Consequently, as the bias window spans the spin-up peak while remaining within the spin-down pseudogap, *I* ^*↑*^ increases nearly linearly with *V* [blue line in Figure 4(a)], whereas *I* ^*↓*^ [blue line in Figure 4(b)] remains considerably smaller, resulting in nearly perfect spin filtering up to high bias.

Including many-body effects leads to a qualitatively different behavior. Within DMFT, the transmission is reduced and broadened as a consequence of the finite quasiparticle lifetime encoded in Im­[Σ^σ*r*^(*E*)]. As the bias increases, this damping suppresses the coherent current [green line in Figure 4(a)], particularly in the spin-up channel, leading to a deviation from the DFT prediction. In contrast, in the spin-down channel, the bias window initially remains within the transmission pseudogap. Only once the spin-down conduction peak, located at *E* – *E*_F_ ≈ 0.4 eV, enters the bias window (i.e., for *V* ∼ 0.8 V) would we expect a similar suppression of the coherent current.

At *V* ≳ 0.25 V, an additional incoherent contribution to the current emerges, represented by the black line in Figure 4. This grows rapidly with bias and becomes comparable to the coherent one. Its onset coincides with the increase of Im­[Σ^σ*r*^(*E*)] at the Fermi level and with the deviation of Σ^σ<^(*E*) from the fluctuation–dissipation relation, linking it directly to the breakdown of quasiparticle coherence in the spectral function.

The effect is particularly pronounced in the spin-down channel, where the differential conductance d*I* ^*↓*^/d*V* exhibits a marked increase once the bias window overlaps with the spin-down states [Figure 1(d)]. This behavior reflects the activation of inelastic scattering channels that are absent in a purely coherent picture. Consequently, a description based solely on the band structure, as typically employed in the modeling of spintronic devices, is insufficient to capture the transport response.

The rapid increase in the differential conductance provides a clear experimental signature of the onset of correlation-driven inelastic transport in the hot-correlated electrons. It can be directly probed through inelastic tunneling spectroscopy using a spin-polarized tip, complementing nano-ARPES measurements. Moreover, the growing incoherent contribution leads to a reduction of the current’s spin-polarization (see Sec. S11 of the SI). While this effect remains moderate in Fe_4_GeTe_2_, the mechanism is fundamental and can become significantly more pronounced in other systems (for instance, Co layers),^27^ where it may ultimately suppress spin-polarization altogether.

Microscopically, the dominant inelastic scattering processes are schematically illustrated in Figure 4(c),(d). Specifically, a spin-up electron injected from the left electrode can scatter off an electron in the spin-down states of Fe_4_GeTe_2_, creating an electron–hole pair excitation that dissipates part of the carrier’s energy. This process becomes active when the applied bias is sufficiently large to allow electron–hole excitations across the spin-down pseudogap, providing an inelastic channel through which a spin-up electron transfers part of its energy before tunneling into an empty state in the right-hand lead [Figure 4(c)]. Similarly, a spin-down electron in the conduction band can exchange energy with spin-up electron–hole excitations, thereby contributing to the spin-down current [Figure 4(d)]. These inelastic processes are similar to those identified in models of inelastic tunneling spectroscopy of magnetic adatoms,^57^ where they typically occur at meV energy scales characteristic of adatoms’ spin states. In contrast, they have so far been largely overlooked in ferromagnets, such as the present system, where they are activated at significantly larger bias voltages, reflecting features of the electronic structure, and can play a central role in shaping bias-driven spin transport in correlated magnetic junctions.

While our calculations rely on several fundamental and practical approximations (see Sections S3-D and S5 of the SI), the physical mechanisms identified here are expected to be robust. Their origin lies in the nonequilibrium redistribution of electronic occupations induced by finite bias, which enhances many-body scattering and is already captured within a relatively simple treatment of the dynamical correlations. More sophisticated theoretical approaches may modify the quantitative strength of this effect but not its nature. For example, if the spin-down pseudogap were slightly smaller than predicted here, the inelastic scattering current would increase, and the onset of the hot-correlated regime would shift to lower bias voltages, thereby even enhancing the bias-induced signatures.

The principal approximation of the present approach is the use of DMFT, which assumes a local electronic self-energy. Consequently, the momentum dependence of the self-energy and the associated nonlocal spin fluctuations are neglected. In particular, this excludes scattering from long-wavelength collective spin excitations (magnons), which could provide additional low-energy scattering channels and further reduce quasiparticle coherence. Furthermore, although here we focused on a collinear ferromagnetic state, Fe_4_GeTe_2_ has a rich magnetic phase diagram^58^ in which noncollinear magnetic textures may arise under specific temperature, field, thickness, and structural conditions.^59–61^ The local many-body mechanism identified here is nevertheless expected to remain valid also in these magnetic phases, while bias-induced scattering with nonlocal spin fluctuations may modify the magnetic texture. Addressing these effects, however, represents an outstanding challenge beyond the already advanced first-principles finite-bias approach developed here.

In summary, our results show that inelastic electron scattering in an Fe_4_GeTe_2_ monolayer under finite bias drives a genuine nonequilibrium regime, which we term the "hot-correlated electron" regime. In this regime, enhanced correlations manifest as incoherent features in both the excitation spectrum and conductance. They are experimentally accessible via nano-ARPES and inelastic tunneling spectroscopy, providing direct routes to verify our predictions. Similar findings are expected across other Fe_*n*_Ge­(Ga)­Te_2_ compounds, owing to their comparable electronic structures.^47^

Beyond their fundamental significance, these effects have direct implications for the current-induced magnetization dynamics. In particular, the emergence of incoherent transport and the associated modification of spin polarization at finite bias are expected to affect both the magnitude and angular dependence of spin-transfer torques used to switch magnetic layers via spin-polarized currents with potential implications for spintronic memory technologies. More broadly, our work establishes voltage-driven electronic correlations as a key factor shaping spin-dependent transport under operating conditions and provides a pathway to investigate nonequilibrium many-body phenomena in realistic device geometries.

## Supplementary Material



## Notes

Material crystal structure; Device setup; Theoretical framework (including: Steady-state NEGF formalism, Projection into the correlated subspace, Dynamical mean-field theory, Charge current); Computational details; Treatment of the static contribution to the DMFT self-energy; Comparison of the electronic structures of the bulk, isolated monolayer, and transport junction; PDOS analysis of the orbital contribution to coherent transport; Orbital occupation and magnetic moments; Spectral function: DFT versus DMFT; Spectral function for negligible Fe_4_GeTe_2_–lead hybridization; Current spin-polarization (PDF)

## References

[1] Milloch A., Fabrizio M., Giannetti C. (2024). Mott materials: unsuccessful metals
with a bright future. npj Spintronics.

[2] Chen B., Yang J., Wang H., Imai M., Ohta H., Michioka C., Yoshimura K., Fang M. (2013). Magnetic properties
of layered itinerant electron ferromagnet Fe_3_GeTe_2_. J. Phys. Soc. Jpn..

[3] Verchenko V. Y., Tsirlin A. A., Sobolev A. V., Presniakov I. A., Shevelkov A. V. (2015). Ferromagnetic order, strong magnetocrystalline anisotropy,
and magnetocaloric effect in the layered telluride Fe_3–*δ*
_GeTe_2_. Inorg.
Chem..

[4] Seo J. (2020). Nearly
room temperature ferromagnetism in a magnetic metal-rich van
der Waals metal. Sci. Adv..

[5] Zhang G., Guo F., Wu H., Wen X., Yang L., Jin W., Zhang W., Chang H. (2022). Above-room-temperature
strong intrinsic
ferromagnetism in 2D van der Waals Fe_3_GaTe_2_ with
large perpendicular magnetic anisotropy. Nat.
Commun..

[6] Xu X. (2020). Signature for non-Stoner
ferromagnetism in the van der Waals ferromagnet
Fe_3_GeTe_2_. Phys. Rev. B.

[7] Zhang Y., Lu H., Zhu X., Tan S., Feng W., Liu Q., Zhang W., Chen Q., Liu Y., Luo X., Xie D., Luo L., Zhang Z., Lai X. (2018). Emergence of Kondo
lattice behavior in a van der Waals itinerant ferromagnet, Fe_3_GeTe_2_. Sci. Adv..

[8] Ghosh S., Ershadrad S., Borisov V., Sanyal B. (2023). Unraveling effects
of electron correlation in two-dimensional Fe_
*n*
_GeTe_2_ (*n* = 3, 4, 5) by dynamical
mean field theory. npj Computational Materials.

[9] Deng Y., Yu Y., Song Y., Zhang J., Wang N. Z., Sun Z., Yi Y., Wu Y. Z., Wu S., Zhu J., Wang J., Chen X. H., Zhang Y. (2018). Gate-tunable room-temperature ferromagnetism
in two-dimensional Fe_3_GeTe_2_. Nature.

[10] Fei Z., Huang B., Malinowski P., Wang W., Song T., Sanchez J., Yao W., Xiao D., Zhu X., May A. F., Wu W., Cobden D. H., Chu J.-H., Xu X. (2018). Two-dimensional itinerant
ferromagnetism in atomically thin Fe3GeTe2. Nat. Mater..

[11] May A. F., Ovchinnikov D., Zheng Q., Hermann R., Calder S., Huang B., Fei Z., Liu Y., Xu X., McGuire M. A. (2019). Ferromagnetism near
room temperature in the cleavable
van der Waals crystal Fe_5_GeTe_2_. ACS Nano.

[12] Silinskas M., Senz S., Gargiani P., Ruiz A. M., Kalkofen B., Kostanovski I., Mohseni K., Baldoví J. J., Meyerheim H. L., Parkin S. S. P., Bedoya-Pinto A. (2024). Self-intercalation
as origin of high-temperature ferromagnetism in epitaxially grown
Fe_5_GeTe_2_ thin films. Phys.
Rev. Lett..

[13] Roemer R. (2024). Unraveling the electronic
structure and magnetic transition evolution
across monolayer, bilayer, and multilayer ferromagnetic Fe_3_GeTe_2_. npj 2D Mater. Appl..

[14] Roemer R., Liu C., Zou K. (2020). Robust ferromagnetism
in wafer-scale monolayer and
multilayer Fe_3_GeTe_2_. npj
2D Mater. Appl..

[15] Min K.-H., Lee D. H., Choi S.-J., Lee I.-H., Seo J., Kim D. W., Ko K.-T., Watanabe K., Taniguchi T., Ha D. H., Kim C., Shim J. H., Eom J., Kim J. S., Jung S. (2022). Tunable spin
injection and detection
across a van der Waals interface. Nat. Mater..

[16] Wang Z., Sapkota D., Taniguchi T., Watanabe K., Mandrus D., Morpurgo A. F. (2018). Tunneling spin valves
based on Fe_3_GeTe_2_/hBN/Fe_3_GeTe_2_ van der Waals heterostructures. Nano
Lett..

[17] Lin H., Yan F., Hu C., Lv Q., Zhu W., Wang Z., Wei Z., Chang K., Wang K. (2020). Spin-valve effect in Fe_3_GeTe_2_/MoS_2_/Fe_3_GeTe_2_ van
der Waals heterostructures. ACS Appl. Mater.
Interfaces..

[18] Zhu W. (2021). Large tunneling magnetoresistance
in van der Waals ferromagnet/semiconductor
heterojunctions. Adv. Mater..

[19] Zhu W., Zhu Y., Zhou T., Zhang X., Lin H., Cui Q., Yan F., Wang Z., Deng Y., Yang H., Zhao L., Žutić I., Belashchenko K. D., Wang K. (2023). Large and tunable magnetoresistance
in van der Waals ferromagnet/semiconductor junctions. Nat. Commun..

[20] Zheng Y., Ma X., Yan F., Lin H., Zhu W., Ji Y., Wang R., Wang K. (2022). Spin filtering
effect in all-van
der Waals heterostructures with WSe_2_ barriers. npj 2D Mater. Appl..

[21] Jin W., Zhang G., Wu H., Yang L., Zhang W., Chang H. (2023). Room-temperature and
tunable tunneling magnetoresistance in Fe_3_GaTe_2_-based 2D van der Waals heterojunctions. ACS
Appl. Mater. Interfaces.

[22] Pan H., Singh A. K., Zhang C., Hu X., Shi J., An L., Wang N., Duan R., Liu Z., Parkin S. S. P., Deb P., Gao W. (2024). Room-temperature tunable tunneling
magnetoresistance in Fe_3_GaTe_2_/WSe_2_/Fe_3_GaTe_2_ van der Waals heterostructures. InfoMat.

[23] Su Y., Li X., Zhu M., Zhang J., You L., Tsymbal E. Y. (2021). Van der
Waals multiferroic tunnel junctions. Nano Lett..

[24] Li X., Lü J.-T., Zhang J., You L., Su Y., Tsymbal E. Y. (2019). Spin-dependent
transport in van der Waals magnetic
tunnel junctions with Fe_3_GeTe_2_ electrodes. Nano Lett..

[25] Zhu Y., Chi B., Jiang L., Guo X., Yan Y., Han X. (2023). Large tunneling
electroresistance, tunneling magnetoresistance, and regulatable negative
differential conductance in a van der Waals antiferroelectric multiferroic
tunnel junction. Phys. Rev. Appl..

[26] Davoudiniya M., Sanyal B. (2024). Efficient spin filtering
through Fe4GeTe2-based van
der Waals heterostructures. Nanoscale Adv..

[27] Nell D., Radonjic M. M., Rungger I., Chioncel L., Sanvito S., Droghetti A. (2026). Nonequilibrium
quantum transport framework for spintronic
devices with dynamical correlations. Phys. Rev.
B.

[28] Metzner W., Vollhardt D. (1989). Correlated
lattice fermions in *d* = *∞* dimensions. Phys. Rev. Lett..

[29] Georges A., Kotliar G., Krauth W., Rozenberg M. J. (1996). Dynamical
mean-field theory of strongly correlated fermion systems and the limit
of infinite dimensions. Rev. Mod. Phys..

[30] Stefanucci, G. ; van Leeuwen, R. Nonequilibrium Many-Body Theory of Quantum Systems: A Modern Introduction; Cambridge University Press, 2013.

[31] Datta, S. Electronic Transport in Mesoscopic Systems; Cambridge University Press, 1995.

[32] Rungger, I. ; Droghetti, A. ; Stamenova, M. In Handbook of Materials Modeling. Vol. 1 Methods: Theory and Modeling; Yip, S. , Andreoni, W. , Eds.; Springer International Publishing, 2019.

[33] Taylor J., Guo H., Wang J. (2001). Ab initio modeling
of quantum transport properties
of molecular electronic devices. Phys. Rev.
B.

[34] Brandbyge M., Mozos J.-L., Ordejón P., Taylor J., Stokbro K. (2002). Density-functional
method for nonequilibrium electron transport. Phys. Rev. B.

[35] Rocha A. R., García-Suárez V. M., Bailey S., Lambert C., Ferrer J., Sanvito S. (2006). Spin and molecular
electronics in
atomically generated orbital landscapes. Phys.
Rev. B.

[36] Nell D., Sanvito S., Rungger I., Droghetti A. (2025). Effect of
dynamical electron correlations on the tunnelling magnetoresistance
of Fe/MgO/Fe(001) junctions. Phys. Rev. B.

[37] Droghetti A., Rungger I. (2017). Quantum transport simulation
scheme including strong
correlations and its application to organic radicals adsorbed on gold. Phys. Rev. B.

[100] Jacob D., Haule K., Kotliar G. (2010). Dynamical mean-field
theory for molecular electronics: Electronic structure and transport
properties. Phys. Rev. B.

[38] Droghetti A., Radonjic M. M., Chioncel L., Rungger I. (2022). Dynamical
mean-field
theory for spin-dependent electron transport in spin-valve devices. Phys. Rev. B.

[39] Chioncel L., Morari C., Östlin A., Appelt W. H., Droghetti A., Radonjić M. M., Rungger I., Vitos L., Eckern U., Postnikov A. V. (2015). Transmission through correlated Cu_
*n*
_CoCu_
*n*
_ heterostructures. Phys. Rev. B.

[40] Morari C., Appelt W. H., Östlin A., Prinz-Zwick A., Schwingenschlögl U., Eckern U., Chioncel L. (2017). Spin-polarized
ballistic conduction through correlated Au – NiMnSb –
Au heterostructures. Phys. Rev. B.

[41] Halder A., Nell D., Sihi A., Bajaj A., Sanvito S., Droghetti A. (2024). Half-metallic
transport and spin-polarized tunneling
through the van der Waals ferromagnet Fe_4_GeTe_2_. Nano Lett..

[42] Abramovitch D. J., Mravlje J., Zhou J.-J., Georges A., Bernardi M. (2024). Respective
roles of electron-phonon and electron-electron interactions in the
transport and quasiparticle properties of SrVO_3_. Phys. Rev. Lett..

[43] Okamoto S. (2007). Nonequilibrium
transport and optical properties of model metal–Mott-insulator–metal
heterostructures. Phys. Rev. B.

[44] Okamoto S. (2008). Nonlinear
transport through strongly correlated two-terminal heterostructures:
a dynamical mean-field approach. Phys. Rev.
Lett..

[45] Arrigoni E., Knap M., von der
Linden W. (2013). Nonequilibrium dynamical mean-field
theory: an auxiliary quantum master equation approach. Phys. Rev. Lett..

[46] Mazzocchi T. M., Werner D., Aichhorn M., Arrigoni E. (2025). Mixed-configuration
approximation for multiorbital systems out of equilibrium. Phys. Rev. B.

[47] Halder A., Nell D., Bajaj A., Sanvito S., Droghetti A. (2026). Spin-dependent
transport in Fe_3_GaTe_2_ and Fe_
*n*
_GeTe_2_ (*n* = 3–5) van der
Waals ferromagnets for magnetic tunnel junctions. 2D Mater..

[48] Schäfer J., Schrupp D., Rotenberg E., Rossnagel K., Koh H., Blaha P., Claessen R. (2004). Electronic
quasiparticle renormalization
on the spin wave energy scale. Phys. Rev. Lett..

[49] Byczuk K., Kollar M., Held K., Yang Y.-F., Nekrasov I. A., Pruschke T., Vollhardt D. (2007). Kinks in the
dispersion of strongly
correlated electrons. Nat. Phys..

[50] Roemer R. (2024). Unraveling the electronic
structure and magnetic transition evolution
across monolayer, bilayer, and multilayer ferromagnetic Fe_3_GeTe_2_. npj 2D Mater. Appl..

[51] Xu Y., Wang Y.-C., Jin X., Liu H., Liu Y., Song H., Tian F. (2024). Mechanism of magnetic
phase transition
in correlated magnetic metal: insight into itinerant ferromagnet Fe_3–*δ*
_GeTe_2_. Communications Physics.

[52] Curcio D., Jones A. J. H., Muzzio R., Volckaert K., Biswas D., Sanders C. E., Dudin P., Cacho C., Singh S., Watanabe K., Taniguchi T., Miwa J. A., Katoch J., Ulstrup S., Hofmann P. (2020). Accessing
the spectral function in a current-carrying device. Phys. Rev. Lett..

[53] Hofmann P. (2021). Accessing
the spectral function of in operando devices by angle-resolved photoemission
spectroscopy. AVS Quantum Science.

[54] Curcio D. (2023). Current-driven insulator-to-metal
transition without Mott breakdown
in Ca_2_RuO_4_. Phys. Rev.
B.

[55] Meir Y., Wingreen N. S. (1992). Landauer formula
for the current through an interacting
electron region. Phys. Rev. Lett..

[56] Ferretti A., Calzolari A., Di Felice R., Manghi F. (2005). First-principles theoretical
description of electronic transport including electron-electron correlation. Phys. Rev. B.

[57] Schweflinghaus B., dos Santos Dias M., Costa A. T., Lounis S. (2014). Renormalization of
electron self-energies via their interaction with spin excitations:
A first-principles investigation. Phys. Rev.
B.

[58] Hasan M. N., Salehi N., Sorgenfrei F., Delin A., Di Marco I., Bergman A., Pereiro M., Thunström P., Eriksson O., Karmakar D. (2025). Dynamical electronic
correlations
and chiral magnetism in the van der Waals magnet Fe_4_GeTe_2_. Phys. Rev. B.

[59] Wang H. (2023). Interfacial engineering of ferromagnetism in wafer-scale
van der
Waals Fe_4_GeTe_2_ far above room temperature. Nat. Commun..

[60] Pal R., Pal B., Mondal S., Sharma R. O., Das T., Mandal P., Pal A. N. (2024). Spin-reorientation
driven emergent phases and unconventional
magnetotransport in quasi-2D vdW ferromagnet Fe_4_GeTe_2_. npj 2D Mater. Appl..

[61] Mondal S., Khan N., Mishra S. M., Satpati B., Mandal P. (2021). Critical behavior
in the van der Waals itinerant ferromagnet Fe_4_GeTe_2_. Phys. Rev. B.

