# Supplementary material for: Bias-Driven Hot-Correlated Electrons in the Two-Dimensional Ferromagnet Fe4GeTe2
Source: Nano Lett. 2026 Sep 9;26(37):12316–22. doi: 10.1021/acs.nanolett.6c02700 (PMC13614302; doi:10.1021/acs.nanolett.6c02700)
Supplement: Supplementary file 1 [file nl6c02700_si_001.pdf]

Supporting Information:
Bias-driven hot-correlated electrons in the two-dimensional ferromagnet Fe_4GeTe_2

Declan Nell,[1] Stefano Sanvito,[1] and Andrea Droghetti[2,*]

[1] *School of Physics and CRANN Institute, Trinity College Dublin, The University of Dublin, Dublin D02 PN40, Ireland*

[2] *Department of Molecular Sciences and Nanosystems, Ca' Foscari University of Venice, via Torino 155, 30170, Mestre, Venice, Italy*

S1. MATERIAL CRYSTAL STRUCTURE

Bulk Fe_4GeTe_2 crystallizes in the R$\bar{3}$m space group (No. 166), whose primitive unit cell is rhombohedral. For layered R$\bar{3}$m materials, however, the hexagonal conventional cell instead of the rhombohedral primitive unit cell is commonly used in quantum-transport calculations. This crystallographically equivalent representation contains three primitive cells and has the crystallographic c axis perpendicular to the layers.

Each Fe_4GeTe_2 layer contains four Fe atoms occupying two crystallographically inequivalent sites, denoted Fe1 and Fe2, which form two Fe–Fe dumbbells. The Ge atom contributes to the in-plane coordination network, while the Te atoms terminate the top and bottom surfaces of each layer.

S2. DEVICE SET-UP

Our prototypical device, shown in Fig. S1, consists of an Fe_4GeTe_2 monolayer connected to two semi-infinite metallic electrodes in a two-probe setup, identical to that considered in Ref. [1], where the complete device geometry and atomic positions are reported.

Transport occurs perpendicular to the monolayer, along the z direction, while periodic boundary conditions are applied in the plane of the layer. Consequently, the electronic states are characterized by the transverse wave vector $\boldsymbol{k} = (k_x, k_y)$ in the corresponding two-dimensional hexagonal Brillouin zone (BZ).

The device is partitioned into left (L) and right (R) leads and a central region, represented within a numerical atomic orbital basis set [2]. Electronic correlations are included only in the central (scattering) region, while the electrodes are treated at the effective single-particle level [3–5], as is customary in quantum transport. Specifically, in the present work the electrodes are described within Kohn–Sham DFT.

The electrodes consist of Au atoms described by a single $6s$ basis orbital. In practice, this provides a generic metallic electrode and represents a common approximation in quantum-transport simulations (e.g., Refs. [6, 7]). To account for interface effects, the central region explicitly includes several atomic layers of each electrode. Consequently, the formal lead/central-region boundary is not located at the physical Fe_4GeTe_2/Au interface, but several layers inside the electrodes, where the electronic structure has already converged to its bulk form. This ensures that the electrodes act as semi-infinite reservoirs while all interface effects are treated explicitly within the central region.

In equilibrium, the leads are maintained at their local chemical potentials, $\mu_{\mathrm{L,R}}$, which define a common Fermi energy, $E_{\mathrm{F}} \equiv \mu_{\mathrm{L}} = \mu_{\mathrm{R}}$. In contrast, a bias voltage, V, shifts the local chemical potentials, $\mu_{\mathrm{L(R)}} = E_{\mathrm{F}} \pm eV/2$, and drives the system out of equilibrium, inducing current flow.

S3. THEORETICAL FRAMEWORK

This section summarizes the theoretical and computational framework, which combines DFT, DMFT, and the NEGF formalism. The framework is implemented in the SMEAGOL quantum transport package [8], which is interfaced

* andrea.droghetti@unive.it

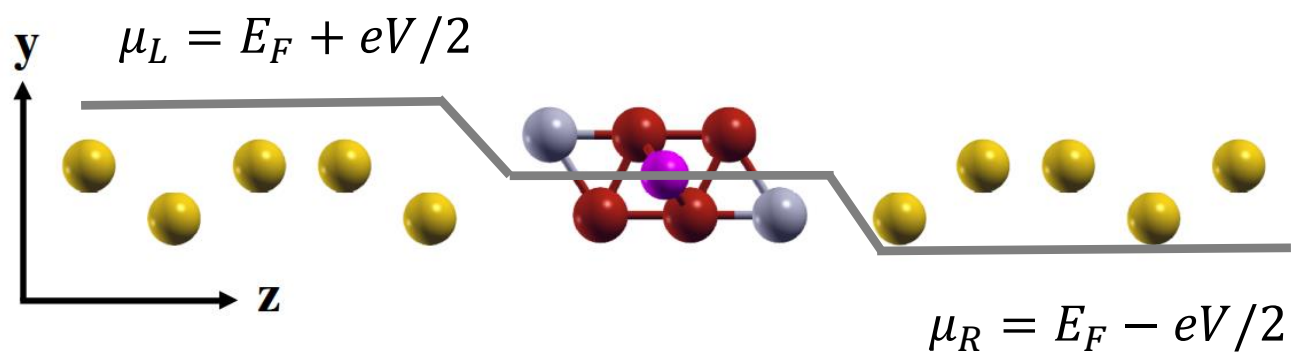

FIG. S1. Atomic structure of the Fe_4GeTe_2 monolayer device, where Fe, Te, Ge, and electrode atoms are shown as red, gray, magenta, and gold spheres, respectively. The schematic potential profile under an applied bias is superimposed across the device, with the L (R) electrode's chemical potential, $\mu_{\mathrm{L(R)}} = E_{\mathrm{F}} \pm eV/2$.

with the SIESTA DFT code [2]. Additional details beyond those presented here can be found in Ref. [9].

A. Steady-state NEGF formalism

The spin (σ)-, energy (E)- and $\boldsymbol{k}$-dependent NEGF of the device's central region is written in a 2×2 block-matrix form [10–12]

$$G^{\sigma}(E,\boldsymbol{k}) = \begin{pmatrix} G^{\sigma\, r}(E,\boldsymbol{k}) & G^{\sigma\, K}(E,\boldsymbol{k}) \\ 0 & G^{\sigma\, a}(E,\boldsymbol{k}) \end{pmatrix}. \tag{1}$$

Here,

$$\begin{gathered} G^{\sigma\, r}(E,\boldsymbol{k}) = \big[(E+i\eta)S(\boldsymbol{k}) - H^{\sigma}_{\mathrm{KS}}(\boldsymbol{k}) - \Sigma^{\sigma\, r}(E,\boldsymbol{k})\big]^{-1}_{\eta\to 0} \\ G^{\sigma\, a}(E,\boldsymbol{k}) = [G^{\sigma\, r}(E,\boldsymbol{k})]^{\dagger}, \\ G^{\sigma\, K}(E,\boldsymbol{k}) = 2G^{\sigma\, <(>)} \underset{(-)}{+} [G^{\sigma\, r}(E,\boldsymbol{k}) - G^{\sigma\, a}(E,\boldsymbol{k})] \end{gathered}$$

denote the retarded (r), advanced (a), and Keldysh (K) components, respectively, where the lesser/greater Green's functions are given by

$$G^{\sigma\, \lessgtr}(E,\boldsymbol{k}) = G^{\sigma\, r}(E,\boldsymbol{k})\, \Sigma^{\sigma\, \lessgtr}(E,\boldsymbol{k})\, G^{\sigma\, a}(E,\boldsymbol{k}). \tag{2}$$

These components satisfy the identity

$$G^{\sigma\, r}(E,\boldsymbol{k}) - G^{\sigma\, a}(E,\boldsymbol{k}) = G^{\sigma\, >}(E,\boldsymbol{k}) - G^{\sigma\, <}(E,\boldsymbol{k}). \tag{3}$$

$H^{\sigma}_{\mathrm{KS}}(\boldsymbol{k})$ and $S(\boldsymbol{k})$ are the DFT Kohn-Sham (KS) Hamiltonian and the orbital overlap matrix of the central region, respectively. The parameter η is a positive infinitesimal.

$\Sigma^{\sigma}(E,\boldsymbol{k})$ denotes the self-energy and comprises contributions from the leads' self-energies, $\Sigma^{\sigma}_{\mathrm{L,R}}(E,\boldsymbol{k})$, describing the hybridization of the central region with the L(R) lead, and the many-body self-energy $\Sigma^{\sigma}_{\mathrm{MB}}(E,\boldsymbol{k})$, accounting for electron-electron interactions. These have the same block form as G^{σ} and can be decomposed into retarded, $\Sigma^{\sigma\, r}(E,\boldsymbol{k})$, advanced, $\Sigma^{\sigma\, a}(E,\boldsymbol{k})$, and lesser/greater, $\Sigma^{\sigma\, (\lessgtr)}(E,\boldsymbol{k})$ components. Therefore $\Sigma^{\sigma\, r/a(\lessgtr)}(E,\boldsymbol{k}) = \Sigma^{\sigma\, r/a(\lessgtr)}_{\mathrm{L}}(E,\mathbf{k}) + \Sigma^{\sigma\, r/a(\lessgtr)}_{\mathrm{R}}(E,\mathbf{k}) + \Sigma^{\sigma\, r/a(\lessgtr)}_{\mathrm{MB}}(E)$.

H^{σ}_{KS}, $S(\boldsymbol{k})$, the NEGFs and the self-energy components are all $N\times N$ matrices, where N is the number of basis orbitals in the device central region.

The spectral function is defined as

$$A^{\sigma}(E,\boldsymbol{k}) = i\big[G^{\sigma\, r}(E,\boldsymbol{k}) - G^{\sigma\, a}(E,\boldsymbol{k})\big], \tag{4}$$

whose integral over the Brillouin zone yields the DOS.

In the absence of the many-body self-energy, the above equations reduce to the standard so-called DFT+NEGF method [8, 13, 14], which provides a static mean-field description of the interaction through the Hartree and exchange-correlation potentials.

Since the leads are assumed to remain in local equilibrium, their lesser and greater self-energies can be expressed using the fluctuation-dissipation theorem [5],

$$\begin{aligned}\Sigma^{\sigma\,<}_{\mathrm{L(R)}}(E,\boldsymbol{k}) =& i f_{\mathrm{L(R)}}(E)\Gamma^{\sigma}_{\mathrm{L(R)}}(E,\boldsymbol{k})\\ \Sigma^{\sigma\,>}_{\mathrm{L(R)}}(E,\boldsymbol{k}) =& i\big[f_{\mathrm{L(R)}}(E)-1\big]\Gamma^{\sigma}_{\mathrm{L(R)}}(E,\boldsymbol{k}),\end{aligned} \tag{5}$$

where $\Gamma^{\sigma}_{\mathrm{L(R)}}(E,\boldsymbol{k}) = i\big[\Sigma^{\sigma\,r}_{\mathrm{L(R)}}(E,\boldsymbol{k}) - \Sigma^{\sigma\,a}_{\mathrm{L(R)}}(E,\boldsymbol{k})\big]$ is the coupling of the L (R) lead to the central region, and $f_{\mathrm{L(R)}}(E)$ is the Fermi function of the corresponding lead, with chemical potential $\mu_{\mathrm{L(R)}}$. Here, $\Sigma^{\sigma\,\lessgtr}_{\mathrm{L(R)}}$ encode information about the out-of-equilibrium in- and outflow of electrons in the central region.

Analogously, for the many-body self-energy we can introduce [15–17]

$$\begin{aligned}\Sigma^{\sigma\,<}_{\mathrm{MB}}(E,\boldsymbol{k}) &= iF^{\sigma}_{\mathrm{MB}}(E)\Gamma^{\sigma}_{\mathrm{MB}}(E,\boldsymbol{k})\;,\\ \Sigma^{\sigma\,>}_{\mathrm{MB}}(E,\boldsymbol{k}) &= i[F^{\sigma}_{\mathrm{MB}}(E)-1]\Gamma^{\sigma}_{\mathrm{MB}}(E,\boldsymbol{k})\;.\end{aligned} \tag{6}$$

Here, $F^{\sigma}_{\mathrm{MB}}(E)$ denotes the occupation matrix of the central device region, and $\Gamma^{\sigma}_{\mathrm{MB}}(E,\boldsymbol{k}) = i\big[\Sigma^{\sigma\,r}_{\mathrm{MB}}(E,\boldsymbol{k}) - \Sigma^{\sigma\,a}_{\mathrm{MB}}(E,\boldsymbol{k})\big]$. In general, it differs from the Fermi function; only in equilibrium does $F^{\sigma}_{\mathrm{MB}}(E)$ reduce to the Fermi function.

B. Projection into the correlated subspace

Electron-electron interactions are included only within the correlated subspace $\mathcal{C}$ spanned by the Fe $3d$ orbitals. This subspace is defined via the projection scheme of Ref. [15].

The KS Hamiltonian is projected onto $\mathcal{C}$ as

$$\bar{H}^{\sigma}_{\mathcal{C}}(\boldsymbol{k}) = \; W(\boldsymbol{k})^{\dagger} H^{\sigma}_{\mathrm{KS}}(\boldsymbol{k}) W(\boldsymbol{k}) \tag{7}$$

where $W(\boldsymbol{k})$ is the transformation matrix defined in Eqs. (17) and (A17) of Ref. [15], which also orthogonalizes the orbitals within $\mathcal{C}$. The projected Hamiltonian $\bar{H}^{\sigma}_{\mathcal{C}}(\boldsymbol{k})$ is then supplemented by a Hubbard-like interaction term.

The NEGF in the $\mathcal{C}$ subspace, $\bar{G}^{\sigma}_{\mathcal{C}}(E,\boldsymbol{k})$, is defined analogously to Eq. (1) as

$$\bar{G}^{\sigma}_{\mathcal{C}}(E,\boldsymbol{k}) = \begin{pmatrix} \bar{G}^{\sigma\;r}_{\mathcal{C}}(E,\boldsymbol{k}) & \bar{G}^{\sigma\;K}_{\mathcal{C}}(E,\boldsymbol{k}) \\ 0 & \bar{G}^{\sigma\;r}_{\mathcal{C}}(E,\boldsymbol{k})^{\dagger} \end{pmatrix}. \tag{8}$$

The projected self-energy is given by

$$\bar{\Sigma}^{\sigma\;r(<)}(E,\boldsymbol{k}) = \bar{\Sigma}^{\sigma\;r(<)}_{\mathrm{L}}(E,\mathbf{k}) + \bar{\Sigma}^{\sigma\;r(<)}_{\mathrm{R}}(E,\mathbf{k}) + \bar{\Sigma}^{\sigma\;r(<)}_{\mathcal{C}}(E,\mathbf{k}) \tag{9}$$

The projected lead self-energies $\mathcal{C}$, $\bar{\Sigma}^{\sigma\;r(<)}_{\mathrm{L(R)}}(E,\mathbf{k})$ are obtained as detailed in Ref. [15] [see Eqs. (71) and (72)].

C. Dynamical mean-field theory

Within DMFT, the many-body self-energy in $\mathcal{C}$ is approximated to be local, i.e., momentum independent and diagonal over the atomic site index:

$$\bar{\Sigma}^{\sigma\;r(<)}_{\mathcal{C}}(E) = \begin{pmatrix} \bar{\Sigma}^{\sigma\;r(<)}_{1}(E) & 0 & \ldots & 0 \\ 0 & \bar{\Sigma}^{\sigma\;r(<)}_{2}(E) & \ldots & 0 \\ 0 & 0 & \ldots & \bar{\Sigma}^{\sigma\;r(<)}_{N_{\mathrm{Fe}}}(E) \end{pmatrix}, \tag{10}$$

where $N_{\rm Fe}$ is the number of Fe atoms in the central region (four in our Fe_4GeTe_2 system), and each block $\bar{\Sigma}_i^{\sigma\ r(<)}(E)$ represents the retarded (lesser) DMFT self-energy for atomic site i within the correlated subspace $\mathcal{C}$.

The DMFT self-energy is obtained self-consistently by mapping the correlated subsystem onto an effective impurity problem via the condition

$$\bar{G}^{\sigma}_{{\rm imp},i}(E) = \bar{G}^{\sigma}_{{\rm loc},i}(E)\,. \tag{11}$$

Here, $\bar{G}^{\sigma}_{{\rm loc},i}(E)$ is the i-th diagonal block of the local NEGF

$$\bar{G}^{\sigma}_{{\rm loc},i}(E) = \frac{1}{\Omega_{\boldsymbol{k}}}\int_{\rm BZ} d\boldsymbol{k}\ \bar{G}^{\sigma}_{\mathcal{C},i}(E,\boldsymbol{k})\,, \tag{12}$$

where $\Omega_{\boldsymbol{k}}$ is the BZ volume, and $\bar{G}^{\sigma}_{{\rm imp},i}(E)$ is the impurity NEGF. The latter satisfies the Dyson equation [12]

$$\bar{G}^{\sigma}_{{\rm imp},i}(E) = \mathcal{G}^{\sigma}_i(E) + \mathcal{G}^{\sigma}_i(E)\bar{\Sigma}^{\sigma}_{{\rm imp},i}(E)\bar{G}^{\sigma}_{{\rm imp},i}(E)\,, \tag{13}$$

where $\mathcal{G}^{\sigma}_i(E)$ is the dynamical mean-field (Weiss field).

The retarded and advanced components of this dynamical mean-field are given by

$$\mathcal{G}^{\sigma\ r(a)}_i(E) = \Big[G^{\sigma\ r(a)}_{{\rm loc},i}(E)^{-1} + \bar{\Sigma}^{\sigma\ r(a)}_i(E)\Big]^{-1}, \tag{14}$$

while the lesser component can be written as

$$\mathcal{G}^{\sigma\ <}_i(E) = \mathcal{G}^{\sigma\ r}_i(E)\Delta^{\sigma\ <}_i(E)\mathcal{G}^{\sigma\ a}_i(E), \tag{15}$$

where $\mathcal{G}^{\sigma\ a}_i(E) = [\mathcal{G}^{\sigma\ r}_i(E)]^{\dagger}$. The lesser hybridization function is defined as

$$\Delta^{\sigma\ <}_i(E) = \bar{G}^{\sigma\ r}_{{\rm loc},i}(E)^{-1}\bar{G}^{\sigma\ <}_{{\rm loc},i}(E)\bar{G}^{\sigma\ a}_{{\rm loc},i}(E)^{-1} - \bar{\Sigma}^{\sigma\ <}_i(E). \tag{16}$$

For the first iteration of the DMFT self-consistent loop, the DMFT self-energies $\bar{\Sigma}^{\sigma\ r}_i(E)$ and $\bar{\Sigma}^{\sigma\ <}_i(E)$ are initialized to zero. At zero bias (equilibrium) $\mathcal{G}^{\sigma\ <}_i(E)$ is related to $\mathcal{G}^{\sigma\ r}_i(E)$ via the fluctuation-dissipation theorem, and the present formulation reduces to layered DMFT as in Refs. [18, 19].

Here, we employ a second-order perturbation-theory impurity solver [19, 20], which provides reliable results for metallic systems [21] and can be naturally extended to nonequilibrium conditions [5].

D. Charge current

The two-terminal steady-state charge current is given by the Meir-Wingreen formula [4]

$$I^{\sigma} = \frac{e}{2h\Omega_{\boldsymbol{k}}}\int d\boldsymbol{k}\int dE\ {\rm Tr}\Big[[\Sigma^{\sigma\ <}_{\rm L}(E,\boldsymbol{k}) - \Sigma^{\sigma\ <}_{\rm R}(E,\boldsymbol{k})]\big[G^{\sigma\ r}(E,\boldsymbol{k}) - G^{\sigma\ a}(E,\boldsymbol{k})\big]\ + i[\Gamma^{\sigma}_{\rm L}(E,\boldsymbol{k}) - \Gamma^{\sigma}_{\rm R}(E,\boldsymbol{k})]G^{\sigma\ <}(E,\boldsymbol{k})\Big]. \tag{17}$$

Using Eq. (3) together with the expressions for the lesser and greater components of the lead and many-body self-energies in Eqs. (5) and (6), the current can be rewritten as the sum of the coherent and incoherent contributions:

$$I^{\sigma} = I^{\sigma}_{\rm C} + I^{\sigma}_{\rm IC}. \tag{18}$$

The coherent contribution reads

$$I^{\sigma}_{\rm C} = \frac{e}{h}\int_{-\infty}^{\infty} dE\ T^{\sigma}(E)\big[f_{\rm L}(E) - f_{\rm R}(E)\big], \tag{19}$$

where $T^{\sigma}(E)$ denotes the transmission coefficient

$$T^{\sigma}(E) = \frac{1}{\Omega_{\boldsymbol{k}}}\int d\boldsymbol{k}\ {\rm Tr}\Big[\Gamma^{\sigma}_{L}(E,\boldsymbol{k})G^{\sigma\ r}(E,\boldsymbol{k})\Gamma^{\sigma}_{R}(E,\boldsymbol{k})G^{\sigma\ a}(E,\boldsymbol{k})\Big]. \tag{20}$$

Equation (19) is the generalization of the Landauer-Büttiker formula to finite bias (see, for example, reference [8, 22, 23]).

The incoherent contribution is

$$
\begin{aligned}
I^{\sigma}_{\mathrm{IC}} =& \frac{e}{2h\Omega_{\boldsymbol{k}}} \int d\boldsymbol{k} \int dE \, \mathrm{Tr}\Big\{ \big[f_{\mathrm{L}}(E) - F^{\sigma}_{\mathrm{MB}}(E)\big] \Gamma^{\sigma}_{\mathrm{MB}}(E)\, G^{\sigma\ a}(E,\boldsymbol{k})\, \Gamma^{\sigma}_{\mathrm{L}}(E,\boldsymbol{k})\, G^{\sigma\ r}(E,\boldsymbol{k}) \Big\} \\
& - \frac{e}{2h\Omega_{\boldsymbol{k}}} \int d\boldsymbol{k} \int dE \, \mathrm{Tr}\Big\{ \big[f_{\mathrm{R}}(E) - F^{\sigma}_{\mathrm{MB}}(E)\big] \Gamma^{\sigma}_{\mathrm{MB}}(E)\, G^{\sigma\ a}(E,\boldsymbol{k})\, \Gamma^{\sigma}_{\mathrm{R}}(E,\boldsymbol{k})\, G^{\sigma\ r}(E,\boldsymbol{k}) \Big\}.
\end{aligned} \tag{21}
$$

In the KS single-particle description, the many-body self-energy is absent, and thus the incoherent contribution vanishes. In this case, the current is given solely by Eq. (19), with $T^{\sigma}(E)$ evaluated using the KS NEGF.

In DMFT, both contributions can be non-negligible. $T^{\sigma}(E)$ incorporates the NEGF with the DMFT self-energy, and represents the transmission for the electronic excitations renormalized in both energy and lifetime by dynamical correlation effects. The incoherent contribution instead captures the impact of inelastic scattering on the transport. Notably, Eq. (21) resembles Eq. (19), but with f^{σ}_{MB} and $\Gamma^{\sigma}_{\mathrm{MB}}$ replacing f_{R} (f_{L}) and $\Gamma^{\sigma}_{\mathrm{R}}$ ($\Gamma^{\sigma}_{\mathrm{L}}$) in the first (second) term of the integrand. This analogy suggests an intuitive picture in which inelastic scattering is effectively represented by an additional "virtual electrode". I_{IC} can then be interpreted as the current flowing from the left lead into this virtual electrode and subsequently emerging into the right lead with modified energy and phase. This picture closely resembles the phenomenological concept of the dephasing probe introduced by Büttiker [24], commonly used to describe the crossover between coherent and incoherent transport in nano-conductors. Eqs. (21) and (19) with the DMFT self-energy provide a quantitative, atomistic, and theoretically grounded framework to address this crossover.

S4. COMPUTATIONAL DETAILS

DFT+NEGF calculations are performed using the same computational details as in Ref. [1].

The DMFT equations are solved in a single-shot fashion after the DFT charge self-consistency has been reached; that is, the DMFT self-energy is not fed back into the DFT charge-density cycle. The orbital-dependent screened Coulomb interaction parameters for the Fe 3d orbitals are expressed in terms of the Slater integrals F^0, F^2, and F^4 [25]. The ratio F^4/F^2 is fixed to the atomic value ≈ 0.625 [26]. The average interaction parameters are defined as $U = F^0$ and $J = (F^2 + F^4)/14$, and in this work, take the values $U = 1.5$ eV and $J = 0.5$ eV.

The local Green's function is computed using a 20×20 $\boldsymbol{k}$-mesh. DMFT calculations are performed directly on the real energy axis with a grid of 10,000 points spanning the range $[-25, 15]$ eV. The resulting converged DMFT self-energy is then used to evaluate the DOS and transmission coefficient on a finer 80×80 $\boldsymbol{k}$-mesh. The orbital occupations are obtained by numerically integrating the DOS up to the Fermi level.

Within our implementation of DMFT, which relies on a perturbative impurity solver, particular care is required in the treatment of the first-order (static) contribution to the self-energy. Since this term is intimately connected to the choice of the double-counting correction, different implementations correspond to different approximations. This aspect is discussed in detail in the following section.

S5. TREATMENT OF THE STATIC CONTRIBUTION TO THE DMFT SELF-ENERGY

The calculations in this Letter and in our previous work, Ref. [1], employ DMFT implementations, which differ in the static first-order contribution to the self-energy, while the dynamical second-order self-energy is evaluated identically in both approaches.

In the zero-bias implementation (hereafter, Imp. 1) used in Ref. [1], the sum of the first-order self-energy contribution and fully localized limit (FLL) double-counting correction was approximated by the effective U potential of the DFT+U formulation of Dudarev *et al.* [27]. In the present implementation (Imp. 2), the first-order self-energy is instead effectively approximated by the DFT (GGA) exchange-correlation potential, which is computationally simpler to handle and leads to faster convergence under finite-bias conditions. Consequently, the second-order self-energy is effectively evaluated using the DFT+U reference Green's function in Imp. 1 and the DFT reference Green's function

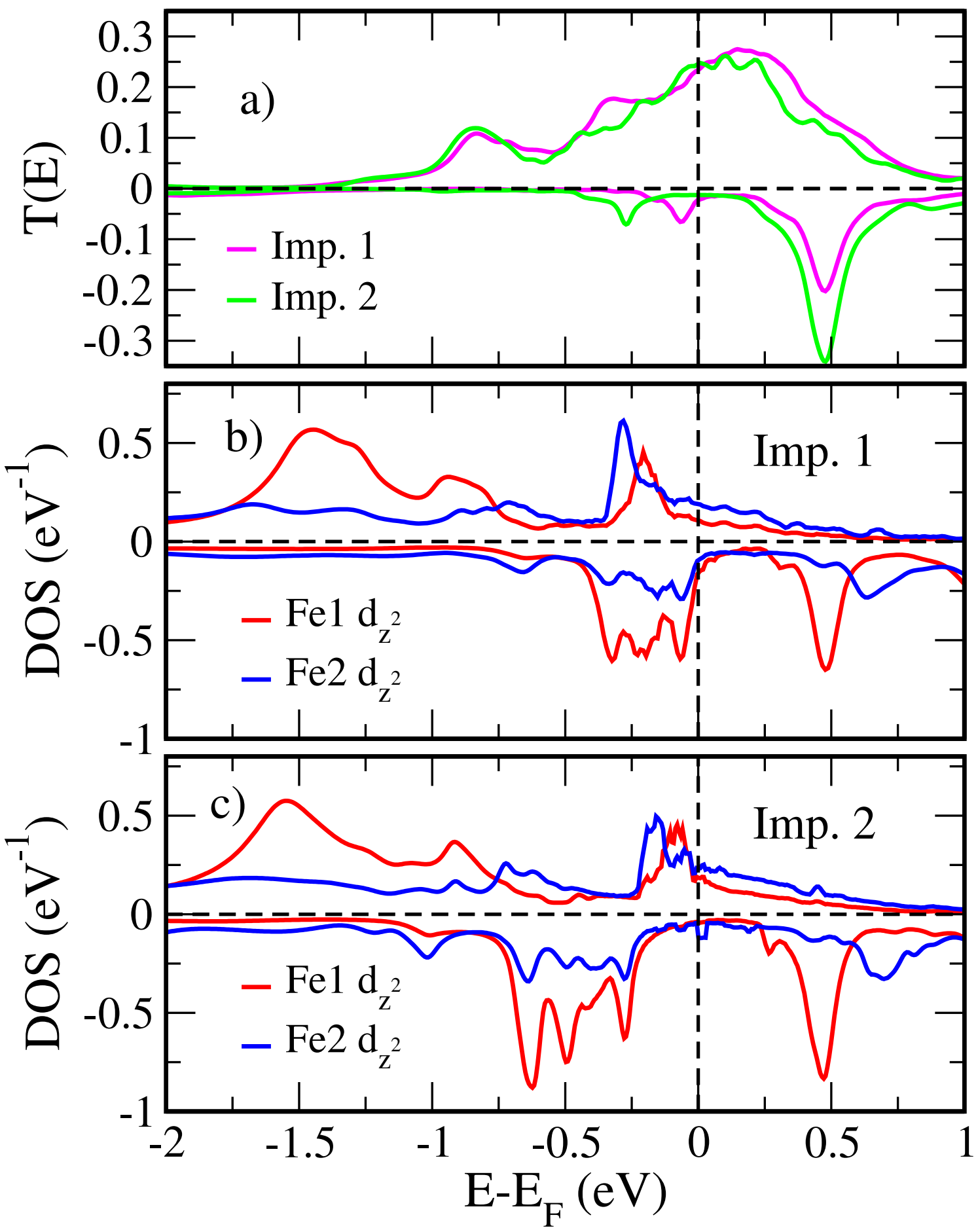

FIG. S2. (a) DMFT zero-bias transmission coefficient calculated using Imp. 1 with $U = 3$ eV and Imp. 2 with $U = 1.5$ eV and $J = 0.5$ eV. (b) DMFT Fe1 and Fe2 d_{z^2}-PDOS obtained with Imp. 1. (c) DMFT Fe1 and Fe2 d_{z^2}-PDOS obtained with Imp. 2.

in Imp. 2.

At a more fundamental level, the difference between the two implementations is closely related to the treatment of the double-counting correction, which remains an open problem in DFT+DMFT transport calculations. Since the double-counting correction acts as an on-site energy shift, different treatments of the first-order self-energy effectively correspond to different choices of this correction. Further details of the two implementations are provided in Ref. [9].

For ferromagnetic transition metals, both approaches give comparable results, albeit for different values of the interaction parameters. Imp. 1 requires larger values of U and J than Imp. 2. This reflects the opposite effects of the static DFT+U potential and the second-order self-energy: the former enhances the exchange splitting already overestimated by DFT, whereas the latter tends to reduce it [20]. When the static DFT+U potential is included, larger values of U are therefore required in the evaluation of the second-order self-energy to compensate for the increase in the exchange splitting.

To analyze the effect of the different interaction parameters in our Fe_4GeTe_2 junction, we compare in Fig. S2 the zero-bias d_{z^2} PDOS and transmission coefficient obtained with the two implementations. In the spin-up channel, the transmission coefficients from Imp. 1 and Imp. 2 are nearly identical. In the spin-down channel, the pseudogap at the Fermi level and the peak around $E - E_F \sim 0.5$ eV are preserved, with only a moderate change in peak width. The main difference in the PDOS is a downward shift of the occupied spin-down d_{z^2} states by approximately 0.2 eV in Imp. 2 compared to Imp. 1 . As a result, the corresponding transmission peak moves from $E - E_F \approx -0.05$ eV to $E - E_F \approx -0.25$ eV, leading to a slightly larger spin-down transport gap. Apart from this quantitative shift, the two implementations produce the same qualitative low-energy electronic structure.

Similarly, we do not expect the bias-induced many-body phenomena reported here to depend qualitatively on the precise choice of the interaction parameters, provided this choice does not alter the underlying nature of the correlated electronic state. Their origin lies in the nonequilibrium redistribution of electronic occupations under finite bias, which enhances many-body scattering irrespective of the specific parametrization of the local interaction. Different values of U and J may nevertheless modify the quantitative strength of this effect. For example, a reduction of the spin-down pseudogap would increase the inelastic scattering current and shift the onset of the hot-correlated regime to lower bias voltages, thereby even enhancing the bias-induced signatures discussed in this work.

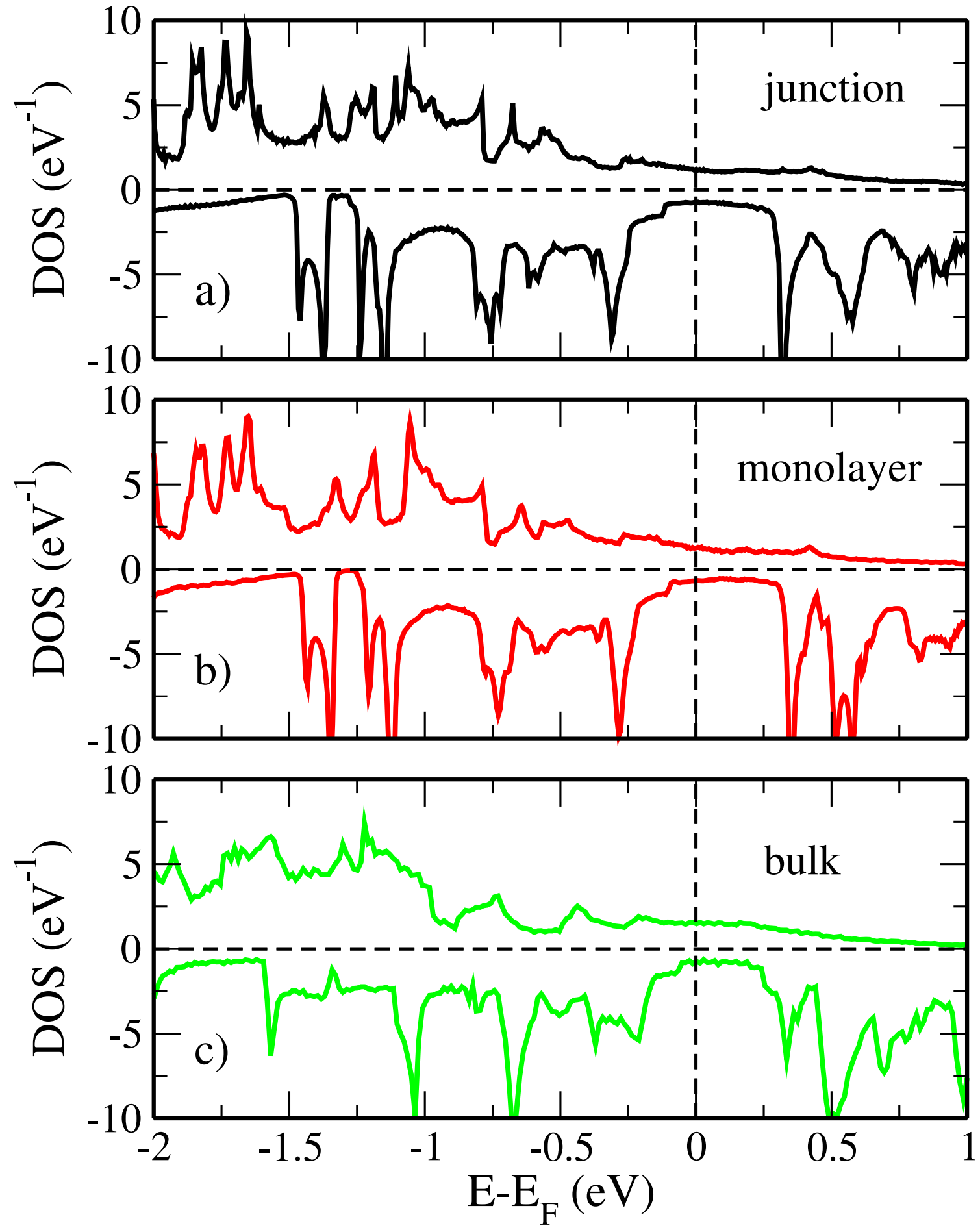

FIG. S3. DFT DOS projected onto the Fe 3d orbitals for (a) the Fe_4GeTe_2 monolayer in the transport junction, (b) an isolated monolayer, and (c) a layer in the bulk crystal. For visual comparison, the DOS of the isolated monolayer has been broadened with a Gaussian function, since in the absence of electrode coupling it consists of sharp δ peaks.

S6. COMPARISON OF THE ELECTRONIC STRUCTURES OF THE BULK, ISOLATED MONOLAYER, AND TRANSPORT JUNCTION

Fig. S3 compares the Fe 3d-PDOS, summed over all 3d orbitals of the inequivalent Fe1 and Fe2 atoms, for (a) the Fe_4GeTe_2 monolayer in the scattering region of the transport junction, (b) an isolated monolayer, and (c) a layer in the bulk crystal, calculated at the DFT level. The isolated monolayer is obtained by placing each electrode 10 Å away from the monolayer, thereby suppressing the electronic coupling between them. The bulk DOS is obtained by replacing the Au electrodes with semi-infinite Fe_4GeTe_2 leads, as described in Refs. [1, 28].

The three spectra are remarkably similar around the Fermi level. In the majority-spin channel, they all exhibit a broad, relatively featureless spectral distribution. In the minority-spin channel, the pseudogap around the Fermi level is present in all three systems and extends over a comparable energy range.

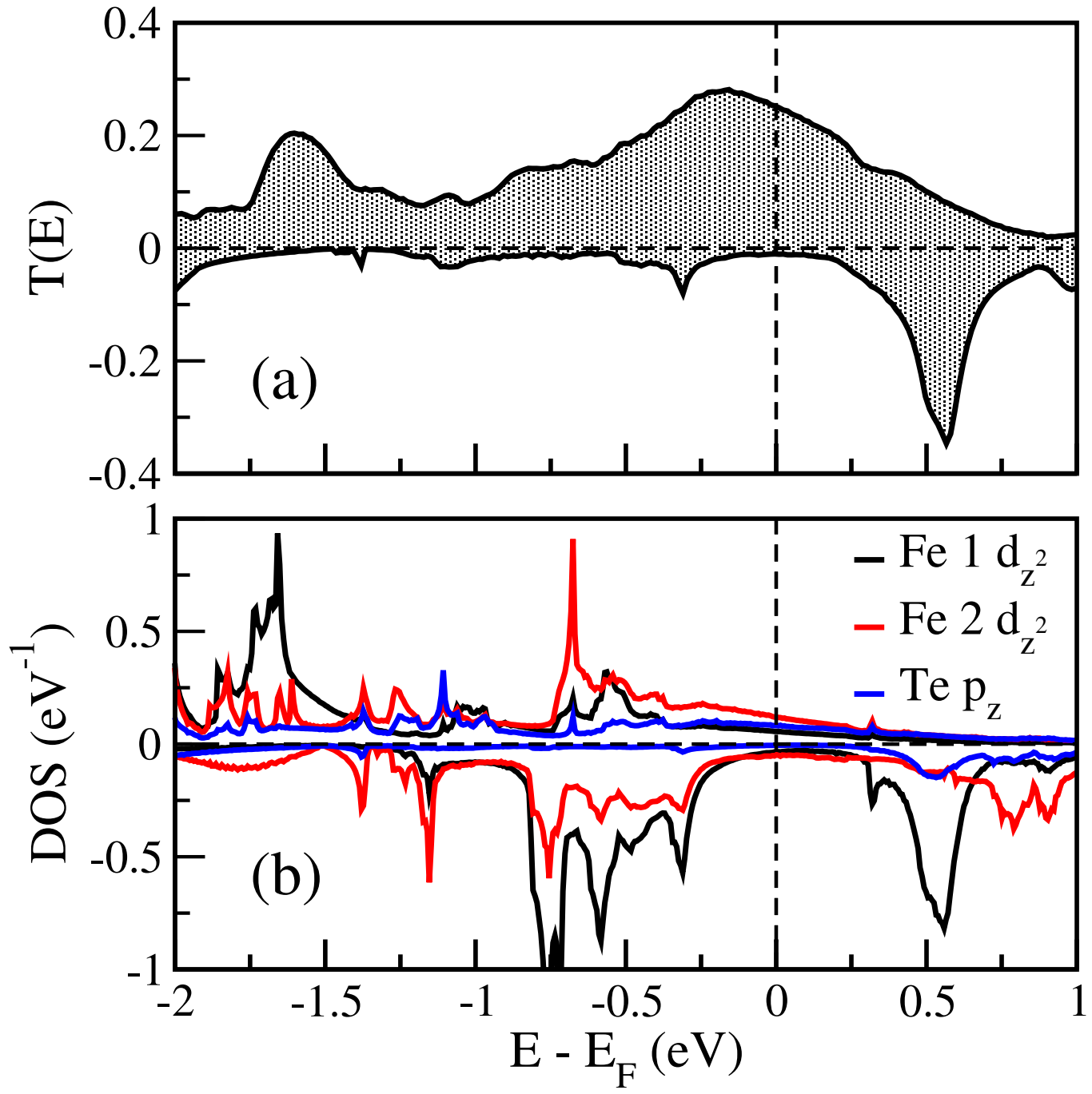

FIG. S4. (a) Zero-bias DFT transmission coefficient for the monolayer. (b) Zero-bias DFT DOS projected over the $3d_{z^2}$ orbitals of Fe1 (black), Fe2 (red) and the $5p_z$ orbital of Te (blue).

The strong similarity between the PDOS of the isolated monolayer and that of the monolayer in the transport junction is preserved even away from the Fermi level, demonstrating the weak effect of the model electrodes on the electronic structure of the monolayer. This effect is nevertheless not entirely negligible. In particular, the $\boldsymbol{k}$-resolved spectral function of the transport junction displays an apparent discontinuity, visible as a light region at the center of the spectrum [Fig. 1(b,c) of the Letter], which is absent in the isolated monolayer (see Sec. S10).

The monolayer in the bulk exhibits some differences compared to the isolated and device case at energies further from the Fermi level, reflecting the effects of interlayer coupling. In particular, the occupied states display a redistribution of spectral weight below $E - E_\mathrm{F} \lesssim -0.3$ eV. However, these differences have little impact on transport, as long as the bias window spans only the energy range from approximately $E - E_\mathrm{F} = -0.3$ to 0.3 eV. Consistent with this picture, our previous work showed that bulk and monolayer junctions exhibit very similar spin-polarized conductances [1].

S7. PDOS ANALYSIS OF THE ORBITAL CONTRIBUTION TO COHERENT TRANSPORT

In Fe_4GeTe_2, the inequivalent Fe1, Fe2, and Te atoms are aligned along the transport direction (z) (see Fig. S1). Consequently, the Fe $3d_{z^2}$–Te $5p_z$ overlap controls the delocalization of the electronic states and, therefore, their coupling to the electrodes and the transmission coefficient, ultimately determining the coherent transport properties of the device. To illustrate this, Fig. S4(b) shows the zero-bias PDOS projected onto the Fe1 $3d_{z^2}$ (black), Fe2 $3d_{z^2}$ (red), and Te $5p_z$ (blue) orbitals, calculated with DFT, while Fig. S4(a) reproduces the zero-bias DFT transmission coefficient, $T^\sigma(E)$, from Fig. 2(a) of the Letter.

For the spin-up channel, the Fe $3d_{z^2}$ and Te $5p_z$ PDOS overlap, meaning that these states strongly hybridize, producing the broad resonance centered at $E - E_\mathrm{F} \approx -0.25$ eV and extending well above the Fermi level. The significant Te $5p_z$ character enables these delocalized states to couple efficiently to the electrodes, resulting in a broad transmission peak that follows the same energy dependence as the PDOS.

The spin-down channel exhibits the pseudogap at the Fermi level, with valence states between -0.8 and -0.25 eV and conduction states around 0.6 eV. Importantly, these states have markedly different orbital character. The conduction states contain substantial Fe $3d_{z^2}$ and Te $5p_z$ contributions, making them delocalized along the transport

direction and giving rise to the large transmission peak at $E - E_\mathrm{F} \approx 0.6$ eV. In contrast, the valence states are almost entirely localized on the Fe atoms, as evidenced by the negligible Te $5p_z$ weight in the corresponding energy range. Their weak coupling to the electrodes therefore results in a valence transmission peak at $E - E_\mathrm{F} \approx -0.25$ eV that is nearly one order of magnitude smaller than the corresponding conduction-band peak.

	atom	$3d_{xy}$ ↑/↓	$3d_{yz}$ ↑/↓	$3d_{z^2}$ ↑/↓	$3d_{xz}$ ↑/↓	$3d_{x^2-y^2}$ ↑/↓	m (μ_B)
DFT	Fe1	0.93/0.33	0.87/0.39	0.93/0.44	0.87/0.39	0.93/0.34	2.59
	Fe2	0.85/0.45	0.85/0.47	0.81/0.48	0.84/0.49	0.84/0.46	1.84
DMFT	Fe1	0.92/0.36	0.84/0.4	0.86/0.45	0.87/0.39	0.91/0.36	2.44
	Fe2	0.81/0.47	0.8/0.49	0.79/0.5	0.8/0.49	0.82/0.46	1.61

TABLE S1. Spin-up/down $3d$ orbital occupations, and magnetic moments, m, of the inequivalent Fe1 and Fe2 atoms, calculated with DFT and DMFT.

S8. ORBITAL OCCUPATION AND MAGNETIC MOMENTS

Table S1 reports the spin-resolved $3d$ orbital occupations and magnetic moments of the inequivalent Fe1 and Fe2 atoms, calculated within both DFT and DMFT. The DMFT calculations produce only modest changes in the orbital occupations and slightly reduce the magnetic moments, from 2.59 to 2.44 μ_B for Fe1 and from 1.84 to 1.61 μ_B for Fe2. Despite this reduction, the DMFT calculations indicate that dynamical correlations do not modify the DFT magnetic state.

Our DFT magnetic moments are in excellent agreement with previous DFT studies [29], although they slightly overestimate the experimental values. The inclusion of dynamical correlations reduces this overestimation. We note, however, that our DMFT results differ quantitatively from those reported in Ref. [29], where the Fe2 moment decreases while the Fe1 moment increases upon including correlations. One possible origin of this discrepancy is that we employ the same interaction U for both inequivalent Fe sites, while Ref. [29] uses site-dependent interaction parameters ($U_\mathrm{Fe1} > U_\mathrm{Fe2}$) obtained from constrained linear-response calculations. Despite these quantitative differences, both calculations lead to relatively modest correlation-induced changes in the magnetic moments.

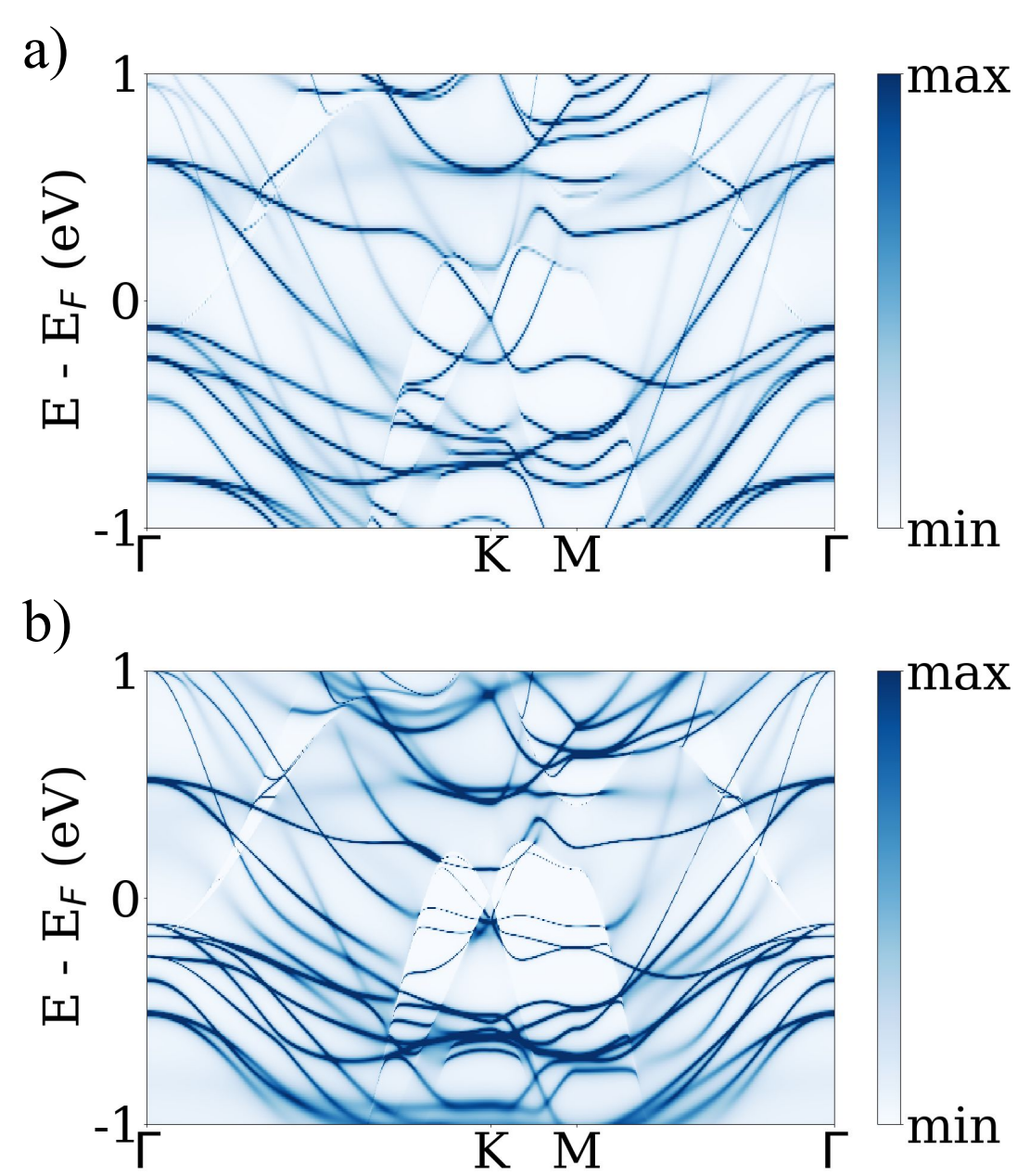

FIG. S5. Zero-bias $\boldsymbol{k}$-resolved Fe $3d$ spectral functions from DFT (a) and DMFT (b).

S9. SPECTRAL FUNCTION: DFT VERSUS DMFT

The spectral function $A(E, \boldsymbol{k})$ at zero bias, calculated within DFT and DMFT, is compared in Fig. S5. Within DFT, characteristic dispersive bands cross E_{F} near the K point [1], with additional broadening arising from hybridization with the leads. Nearly flat bands appear around the Γ point, originating from Fe d_{z^2} orbitals and underpinning the correlated and magnetic character of the system [30].

In contrast, DMFT yields a renormalized spectral function with reduced quasiparticle coherence and enhanced broadening, consistent with the corresponding PDOS. At zero bias, spectral features near E_{F} remain discernible, albeit with reduced sharpness and partial loss of band character.

S10. SPECTRAL FUNCTION FOR NEGLIGIBLE MONOLAYER–LEAD HYBRIDIZATION

Figure S6 shows the DMFT $\boldsymbol{k}$-resolved Fe 3d spectral function at zero-bias calculated after increasing the Fe_4GeTe_2 monolayer–electrode separation to 5 Å. Unlike the results shown in Fig. 1(b) of the Letter and in Fig. S5, the apparent discontinuity visible as a light region at the center of the spectrum is absent. This confirms that the feature originates from the hybridization between the Fe_4GeTe_2 monolayer and the electrodes.

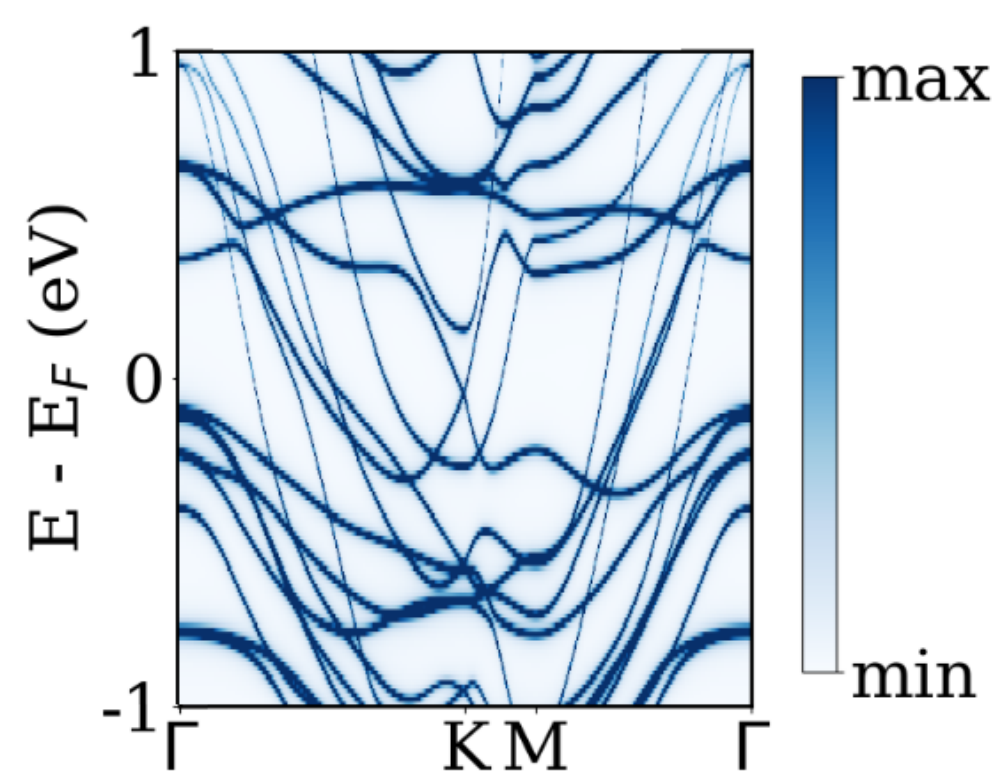

FIG. S6. Zero-bias DMFT $\boldsymbol{k}$-resolved Fe 3d spectral function calculated with the Fe_4GeTe_2 monolayer separated by 5 Å from the electrodes.

S11. CURRENT SPIN POLARIZATION

Figure S7 shows the current spin polarization,

$$\mathrm{SP} = \frac{I^{\uparrow} - I^{\downarrow}}{I^{\uparrow} + I^{\downarrow}}, \tag{22}$$

calculated within DFT and DFT+DMFT (blue and red curves, respectively). At low bias, the current remains almost fully spin-polarized (SP ≈ 0.9). At larger bias, however, dynamical electronic correlations lead to a noticeable reduction of the spin polarization. For instance, at $V = 0.6$ V, the current spin polarization is reduced by approximately 25%. This behavior originates from the correlation-induced enhancement of the minority-spin current, which sets in once the hot-correlated regime is reached at about 0.3 V (see Fig. 4 and the related discussion in the Letter). Although the reduction is sizable, it is not dramatic in Fe_4GeTe_2. Nevertheless, the underlying correlation-driven mechanism is expected to be quite general. Its magnitude depends sensitively on the electronic structure and may therefore become much larger in other systems [9].

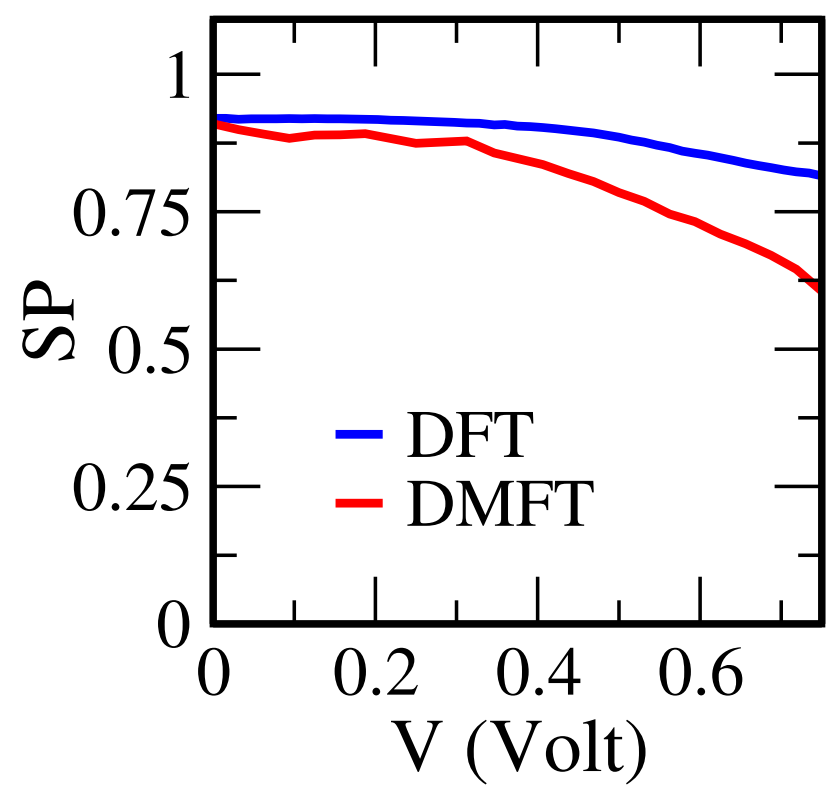

FIG. S7. Current spin polarization as a function of bias, calculated within DFT and DFT+DMFT.

[1] Halder, A.; Nell, D.; Sihi, A.; Bajaj, A.; Sanvito, S.; Droghetti, A. Half-metallic transport and spin-polarized tunneling through the van der Waals ferromagnet Fe_4GeTe_2. *Nano Lett.* **2024**, *24*, 9221.

[2] Soler, J. M.; Artacho, E.; Gale, J. D.; García, A.; Junquera, J.; Ordejón, P.; Sánchez-Portal, D. The SIESTA method for *ab initio* order-N materials simulation. *Journal of Physics: Condensed Matter* **2002**, *14*, 2745–2779.

[3] Haug, H.; Jauho, A. *Quantum Kinetics in Transport and Optics of Semiconductors*; Solid-State Sciences Series; Springer Berlin Heidelberg, 1996.

[4] Meir, Y.; Wingreen, N. S. Landauer formula for the current through an interacting electron region. *Phys. Rev. Lett.* **1992**, *68*, 2512.

[5] Stefanucci, G.; van Leeuwen, R. *Nonequilibrium Many-Body Theory of Quantum Systems: A Modern Introduction*; Cambridge University Press, 2013.

[6] Toher, C.; Filippetti, A.; Sanvito, S.; Burke, K. Self-interaction errors in density-functional calculations of electronic transport. *Phys. Rev. Lett.* **2005**, *95*, 146402.

[7] Nell, D.; Sanvito, S.; Rungger, I.; Droghetti, A. Effect of dynamical electron correlations on the tunnelling magnetoresistance of Fe/MgO/Fe(001) junctions. *Phys. Rev. B* **2025**, *111*, 035133.

[8] Rocha, A. R.; García-Suárez, V. M.; Bailey, S.; Lambert, C.; Ferrer, J.; Sanvito, S. Spin and molecular electronics in atomically generated orbital landscapes. *Phys. Rev. B* **2006**, *73*, 085414.

[9] Nell, D.; Radonjič, M. M.; Rungger, I.; Chioncel, L.; Sanvito, S.; Droghetti, A. Nonequilibrium quantum transport framework for spintronic devices with dynamical correlations. *Phys. Rev. B* **2026**, *113*, 155132.

[10] Rammer, J.; Smith, H. Quantum field-theoretical methods in transport theory of metals. *Rev. Mod. Phys.* **1986**, *58*, 323.

[11] Aligia, A. A. Nonequilibrium magnetotransport through a quantum dot: an interpolative perturbative approach. *Phys. Rev. B* **2006**, *74*, 155125.

[12] Arrigoni, E.; Knap, M.; von der Linden, W. Nonequilibrium dynamical mean-field theory: an auxiliary quantum master equation approach. *Phys. Rev. Lett.* **2013**, *110*, 086403.

[13] Taylor, J.; Guo, H.; Wang, J. *Ab initio* modeling of quantum transport properties of molecular electronic devices. *Phys. Rev. B* **2001**, *63*, 245407.

[14] Brandbyge, M.; Mozos, J.-L.; Ordejón, P.; Taylor, J.; Stokbro, K. Density-functional method for nonequilibrium electron transport. *Phys. Rev. B* **2002**, *65*, 165401.

[15] Droghetti, A.; Rungger, I. Quantum transport simulation scheme including strong correlations and its application to organic radicals adsorbed on gold. *Phys. Rev. B* **2017**, *95*, 085131.

[16] Ness, H.; Dash, L. K.; Godby, R. W. Generalization and applicability of the Landauer formula for nonequilibrium current in the presence of interactions. *Phys. Rev. B* **2010**, *82*, 085426.

[17] Rungger, I.; Droghetti, A.; Stamenova, M. In *Handbook of Materials Modeling. Vol. 1 Methods: Theory and Modeling*; Yip, S., W. Andreoni, W., Eds.; Springer International Publishing, 2019.

[18] Valli, A.; Sangiovanni, G.; Toschi, A.; Held, K. Correlation effects in transport properties of interacting nanostructures. *Phys. Rev. B* **2012**, *86*, 115418.

[19] Droghetti, A.; Radonjič, M. M.; Chioncel, L.; Rungger, I. Dynamical mean-field theory for spin-dependent electron transport in spin-valve devices. *Phys. Rev. B* **2022**, *106*, 075156.

[20] Droghetti, A.; Radonjič, M. M.; Halder, A.; Rungger, I.; Chioncel, L. DFT $+\,\Sigma_2$ method for electron correlation effects at transition metal surfaces. *Phys. Rev. B* **2022**, *105*, 115129.

[21] Janas, D. M.; Droghetti, A.; Ponzoni, S.; Cojocariu, I.; Jugovac, M.; Feyer, V.; Radonjić, M. M.; Rungger, I.; Chioncel, L.; Zamborlini, G.; Cinchetti, M. Enhancing Electron Correlation at a 3*d* Ferromagnetic Surface. *Adv. Mater.* **2023**, *35*, 2205698.

[22] Datta, S. *Electronic Transport in Mesoscopic Systems*; Cambridge University Press, 1995.

[23] Sanvito, S. In *Handbook of Computational Nanotechnology, Vol. 5*; Rieth, M., Schommers, W., Eds.; American Scientific Publishers, 2005.

[24] Büttiker, M. Four-terminal phase-coherent conductance. *Phys. Rev. Lett.* **1986**, *57*, 1761.

[25] Imada, M.; Fujimori, A.; Tokura, Y. Metal-insulator transitions and correlated Metals in *d*-electron systems. *Rev. Mod. Phys.* **1998**, *70*, 1039.

[26] Anisimov, V. I.; Gunnarsson, O. Density-functional calculation of effective Coulomb interactions in metals. *Phys. Rev. B* **1991**, *43*, 7570.

[27] Dudarev, S. L.; Botton, G. A.; Savrasov, S. Y.; Humphreys, C. J.; Sutton, A. P. Electron-energy-loss spectra and the structural stability of nickel oxide: An LSDA+U study. *Phys. Rev. B* **1998**, *57*, 1505–1509.

[28] Halder, A.; Nell, D.; Bajaj, A.; Sanvito, S.; Droghetti, A. Spin-dependent transport in Fe_3GaTe_2 and Fe_nGeTe_2 (n = 3-5) van der Waals ferromagnets for magnetic tunnel junctions. *2D Mater.* **2026**, *13*, 025002.

[29] Ghosh, S.; Ershadrad, S.; Borisov, V.; Sanyal, B. Unraveling effects of electron correlation in two-dimensional Fe_nGeTe_2 ($n = 3, 4, 5$) by dynamical mean field theory. *npj Computational Materials* **2023**, *9*, 86.

[30] Roemer, R. et al. Unraveling the electronic structure and magnetic transition evolution across monolayer, bilayer, and multilayer ferromagnetic Fe_3GeTe_2. *npj 2D Mater. Appl.* **2024**, *8*, 63.
